# The Joint Effects of Habitat Types and Surrounding Landscape Patterns on the Diversity of True Bugs in Southwest China

**DOI:** 10.3390/insects17050497

**Published:** 2026-05-13

**Authors:** Shutong Gao, Zhixing Lu, Xiang Zhang, Qiao Li, Youqing Chen

**Affiliations:** 1 Institute of Highland Forest Science, Chinese Academy of Forestry, Kunming 650224, China; gshuton@163.com (S.G.); luzhixing@caf.ac.cn (Z.L.); m18213456905@163.com (X.Z.); 2 Southwest Survey and Planning Institute of National Forestry and Grassland Administration, Kunming 650031, China; 3 Yunnan Key Laboratory of Breeding and Utilization of Resource Insects, Kunming 650224, China; 4 College of Biodiversity Conservation, Southwest Forestry University, Kunming 650224, China; lqfcb@126.com

**Keywords:** landscape pattern, functional diversity, morphometric traits, insect conservation, Heteroptera

## Abstract

Understanding the drivers of arthropod community diversity is essential for effective biodiversity conservation, particularly in human-dominated landscapes. True bugs (Hemiptera) are morphologically and ecologically diverse, differing in feeding strategy, specialization, and mobility, and thus occupy a wide range of habitats. We sampled true bugs using sweep nets across 257 plots in the Xishuangbanna Biodiversity Priority Area, Southwest China, including natural forests, planted forests, cultivated lands, and complex habitats comprising mosaics of these land-use types. We quantified morphological traits and assessed how habitat type and surrounding landscape structure at multiple spatial scales influence taxonomic and functional diversity. Species richness and abundance were lowest in cultivated lands, whereas functional diversity remained relatively consistent across habitats. Landscape composition and configuration significantly shaped community structure, with effects varying by habitat type and spatial scale. Overall, landscape heterogeneity promoted species richness and abundance, while connectivity and fragmentation showed contrasting effects depending on habitat context. Complex habitats and forests generally supported higher diversity under heterogeneous and well-connected landscapes. These results demonstrate that habitat characteristics and landscape structure jointly regulate true bug diversity, highlighting the need for habitat-specific landscape management that enhances heterogeneity and connectivity in natural and complex habitats while reducing isolation and simplification in human-modified systems.

## Simple Summary

Insect diversity is declining worldwide, especially in human-dominated landscapes, yet the traits that allow species to persist remain unclear. We studied true bugs across 257 sites in a tropical region of Southwest China and measured the morphological traits of collected species. Farmland supported the fewest species and individuals, while forests and mixed habitats maintained higher diversity. Despite these differences, the functional diversity remained broadly similar across habitats. The surrounding landscape also mattered: areas with a greater variety of habitat types supported more species, while the effects of habitat connectivity and fragmentation depended on habitat type. Our results show that both body features and landscape structure shape insect communities, highlighting the importance of maintaining diverse and well-connected habitats to conserve biodiversity.

## 1. Introduction

Human activities have led to habitat degradation, loss, and fragmentation, contributing to a decline in biodiversity across many regions of the world. Efforts to mitigate this trend include the establishment of nature reserves. However, in many instances, nature reserves fail to encompass all habitat types and species [1,2,3]. Furthermore, in human-dominated landscapes, nature reserves are often isolated and fragmented, and a considerable proportion of biodiversity is found in small patches outside these nature reserves [2]. In this regard, cultivated land and agricultural areas often become important targets for conservation efforts [4,5,6,7,8], with linear landscape elements (e.g., field margins, road verges and riverbanks) playing a crucial role in providing secondary habitats for numerous species.

In human-dominated landscapes, biodiversity loss is largely driven by habitat change, which often manifests through alterations in vegetation structure and diversity. Because vegetation complexity underpins arthropod diversity and abundance—by providing sites for settlement, oviposition, resting, concealment, and overwintering—its simplification can have cascading ecological consequences [9,10,11,12]. In spatially heterogeneous agricultural landscapes, different habitat types often exhibit substantial variation in vegetation composition and structure; for example, the vegetation in croplands tends to be more homogenous than that in natural forests. Distinct habitats can offer specialized niches and refuges for different species, thereby fostering the development of varied communities [13,14,15].

However, in human-dominated landscapes, the suitability of a given area for arthropods depends not only on habitat characteristics, but also on the composition and configuration of the landscape at both local and regional scales [14,16,17,18,19]. For example, Montagnana et al. (2021) [20] found that forests with high heterogeneity in surrounding landscape components are more beneficial for the diversity of Hymenoptera than those with low heterogeneity; Torma et al. (2014) [14] found that the isolation of surrounding patches had a significant effect on the diversity of Heteroptera in sand steppe, but this was not observed with other grassland types. The surrounding landscape influences their migration and colonization patterns [21], and adjacent habitats within the local landscape can provide complementary or supplementary resources for certain species. Cross-habitat foraging may link the dynamics of different habitats or landscape elements. Consequently, any community within a given patch is the result of habitat and surrounding landscape matrix filtering. However, most studies have focused solely on the impact of landscape features on biodiversity, with the combined effects of habitat and surrounding landscape details (such as shape complexity and connectivity) on biodiversity remaining largely unexplored.

Arthropods are a crucial component of agricultural landscapes, making significant contributions to biodiversity as well as the function and stability of ecosystems [22,23,24]. Insects, as one of the most diverse arthropod groups, play irreplaceable roles within ecosystems [25,26]. Research has shown a significant decline in insect diversity and biomass over recent decades [27,28], yet, compared to vertebrates and plants, insects often receive less attention in conservation assessments and environmental management activities [3]. True bugs, with their widespread distribution, ease of sampling, and morphological variability, are ideal subjects for community diversity studies [12]. They are the most diverse group among hemimetabolic insects. Their diversity has been shown to be a reliable indicator of overall insect diversity [29]. Importantly, many true bugs are important indicators of biodiversity, effectively reflecting the status of biodiversity and ecosystems [30,31], and are frequently used in conservation assessments [12,29,32]. However, understanding of true bug diversity in tropical and subtropical climates remains preliminary [33].

The study area is part of the Xishuangbanna Priority Areas of Biodiversity Conservation in Yunnan, China, where five nature reserves have been established. The agricultural component of this study area predominantly consists of small-scale farm settlements characterized by numerous, small-sized plots. A mosaic of various habitat types—including natural forests, planted forests, and cultivated lands—forms complex landscape patterns that are markedly different from those of the large-scale agricultural operations previously studied. This study focuses on areas outside of fenced nature reserves, selecting natural forests, planted forests, cultivated land, and complex habitats as representative habitat types for true bug community surveys. The aim is to analyze how different habitat types and landscape structures jointly influence true bug community diversity, trying to answer the question of how to maintain biodiversity outside nature reserves in human-dominated landscapes. Specifically, we seek to address the following questions: (1) How do differences in habitat types affect the species diversity and functional diversity of true bugs? (2) How does landscape structure at different spatial scales influence the species diversity and functional diversity of true bugs? (3) Do the effects of landscape structure on species diversity and functional diversity of true bugs vary across different habitat types? We hypothesize that true bug diversity may be higher in natural forests and mixed habitats compared to farmlands and plantations. Additionally, we hypothesize that the same landscape variables may have differing effects on insect diversity across various habitat types.

## 2. Materials and Methods

### 2.1. Study Area

**Figure 1:**
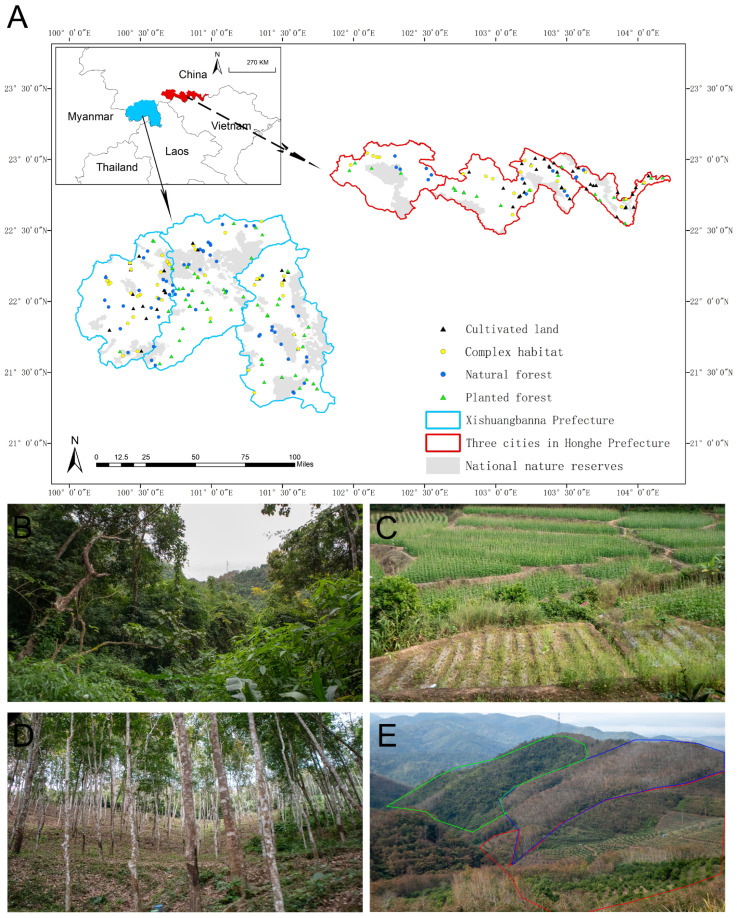
Sampling locations and habitat type representations. (**A**) Depiction of the study area featuring 260 sampling sites, with four habitat types denoted by distinct symbols. (**B**) Natural forest; (**C**) cultivated land; (**D**) planted forest; (**E**) complex habitat.

The research area is located in the southwestern part of Yunnan Province, China, spanning from 99°55′ E to 104°17′ E and from 21°10′ N to 23°08′ N, across Xishuangbanna and three cities within Honghe Prefectures (Figure 1A). Our research area is part of the “Xishuangbanna Priority Conservation Area (42,858 km^2^)”, which includes six national nature reserves, five of which are included in our research area.

Xishuangbanna Prefecture covers an area of 19,124.5 km^2^, which includes two national nature reserves. The region features a tropical monsoon climate and ranges in elevation from 477 m to 2429 m.

In Honghe Prefecture, the counties of Lüchun, Jinping, and Hekou cover a total area of 8105.86 km^2^, including three national nature reserves. These counties are positioned on a low-latitude subtropical plateau, experiencing a moist monsoon climate, with elevational extents from 76.4 m to 3074 m.

Agricultural practices in this region are diverse and traditional, dominated by small-scale farms based on individual family units, resulting in a landscape fragmented into numerous small, distinct patches. Low-altitude (76.4–1000 m) areas primarily cultivate vegetables such as cabbages, corn, and legumes, as well as fruits like bananas and pineapples. While mid-altitude (1000–2000 m) zones are characterized by a mix of crops and predominantly planted forests, such as rubber plantations, high-altitude (2000–3074 m) areas typically feature a mix of natural forests and planted forests, with some cultivated lands. Natural forests are mainly composed of perennial tall trees, such as Fagaceae, Lauraceae, *Trema orientalis*, *Mallotus paniculatus*, and *Macaranga denticulata*. This study focused on four habitat types: natural forests, planted forests, cultivated lands, and complex habitats (Figure 1). A complex habitat refers to a sampling site formed by at least two types of simple habitats from among natural forest, plantation forest, and cultivated land.

### 2.2. Sampling Design and Insect Collection

To obtain landscape diversity data and reduce spatial autocorrelation, the research area was divided into a 10 km × 10 km grid, with 1–2 sites chosen per grid cell. Ultimately, 260 sampling sites were established, including 55 cultivated lands (elevation range: 93–1481 m a.s.l), 69 natural forests (elevation range: 195–2181 m a.s.l), 74 planted forests (elevation range: 194–1687 m a.s.l), and 62 complex habitats (elevation range: 222–2323 m a.s.l). Sampling was conducted twice in the Honghe in July and September, and twice in the Xishuangbanna in August and October. To minimize seasonal bias, each site was sampled once during the same phenological period (the wet season, July–October) when vegetation was fully developed and insect activity was at its peak. Sampling across habitats was interleaved throughout the sampling period to avoid confounding habitat type with sampling date. No site was resampled, and thus our data represent spatial rather than temporal variation. Each sampling team consisted of 3–4 members, totaling seven teams. At each site, three transects, each 100 m in length and 10 m in width, were established, with a minimum distance of 25 m between them. Sweep netting is a common method for collecting arthropods dwelling on plants [14]. In our study, true bugs were collected using 50 cm diameter nets in the shrub and herbaceous layers of different habitats. Each transect was swept at least 150 times, with each sampling session lasting no less than 30 min. During collection, data on longitude, latitude, elevation, habitat type, and predominant vegetation cover at each sampling site were recorded.

### 2.3. Functional Diversity

To calculate the Functional Diversity (FD) index, morphological measurements were taken for all available individuals of all species. The measurement method is detailed in Gao et al. (2024) [34]. Based on these measurements, nine morphometric traits were calculated, covering five functional traits: body size (Body length), dispersal ability (Rel. Forewing length, Hind-Femur shape, Rel. Hind-Femur length) [35,36,37,38,39], feeding resource use (Rel. Rostrum length, Front-Femur shape) [40,41,42,43,44], habitat use (body shape) [45,46,47,48,49], and orientation (Rel. Eye size, Rel. Antenna length) [35,46]. (1) Body length was defined as the average length of male and female individuals, while the (2) body shape is defined as the ratio of body length to body width [45,50]. The lengths of (3) the hind femur, (4) wing, (5) rostrum, and (6) antennae were expressed as relative values to body length [35]. (7) The shape of the hind femur was determined by dividing the length of the hind femur by its width [35], and similarly, (8) the shape of the fore femur was calculated by dividing the length of the fore femur by its width [36]. (9) Eye width was defined as a relative value to head width [46] (see Supplementary Table S1).

Three indices were used to assess the functional diversity of true bugs. The Functional Richness Index (FRic) quantifies the volume of functional space occupied by the community. The Functional Evenness Index (FEve) measures the regularity of abundance distribution within this volume, reflecting the uniformity of species’ functional trait distribution across the ecological space of the community, and indicating the efficiency of resource utilization [51,52]. Functional Divergence (FDiv) reflects the extent to which species in the community deviate from the centroid of the functional space, taking into account their abundance [51]. Lower divergence is observed when the most abundant species are near the centroid of this multidimensional trait space; conversely, high divergence occurs when the most abundant species occupy extreme positions in this space [53].

### 2.4. Estimation of Landscape Features

In this study, landscape indices were calculated at seven spatial scales, within radii of 250 m, 500 m, 1000 m, 1500 m, 2000 m, 3000 m, and 5000 m, using each sampling site as the center. These indices, including the proportion of semi-natural habitat (pSNH), Shannon Landscape Diversity Index (SHDI), Patch Density (PD), Mean Shape Index (MSI), Mean Patch Size (MPS), Contagion Index (CONTAG), and Patch Cohesion Index (COHESION), were employed to quantify the compositional and configurational heterogeneity of the landscape. Landscape data were derived from the 30 m resolution global land cover map GlobeLand30 [54,55]. Landscape indices were calculated using Arc GIS 10.4.1 and Fragstats 4.2.

SHDI represents the diversity and evenness of land cover types within a landscape. The higher the index, the more diverse the landscape, with a variety of land cover types distributed relatively evenly. PD is a direct measure of landscape fragmentation. High patch density often reflects greater fragmentation. MSI is a metric used to describe the shape complexity of patches within a landscape. A higher MSI generally indicates that patches are more irregular or complex in shape. A high MPS indicates that, on average, the patches are large, which suggests less fragmentation and more continuity in the landscape. Both CONTAG and COHESION measure aspects of landscape aggregation, but they are slightly different in focus. While CONTAG measures the overall aggregation of patches of a particular land cover type, COHESION focuses more directly on the spatial connectivity between patches and the extent to which patches are aggregated in the landscape.

### 2.5. Data Analysis

We employed five diversity metrics to characterize the community structure of true bugs: (i) Abundance, quantified as the number of individuals per sample; (ii) Species richness, for which we estimated the richness of True bugs by generating an extrapolation and rarefaction curve utilizing the Chao-1 estimator. These analyses were conducted using the iNEXT software package; (iii) Functional richness (FRic), (iv) Functional evenness (FEve), and (v) Functional divergence (FDiv) were carried out in R 4.4.2, using the “bdFD” function from the “FD” package. This method, by integrating functional trait data and species abundance information, can accurately assess all aspects of community functional diversity.

The normality of the data was assessed using the Shapiro–Wilk test (see Supplementary Table S2), and homogeneity of variances was evaluated using the Levene test (see Supplementary Table S3). Based on these assessments, analysis of variance (ANOVA) and appropriate post hoc tests (LSD test) were applied to compare the functional diversity indices of true bugs across different habitat types. Non-parametric statistics, including the Kruskal–Wallis test and Mann–Whitney U-test with Bonferroni corrections for multiple comparisons, were utilized to compare species richness and abundance of true bugs among the habitat types. Sample-size-based rarefaction and extrapolation sampling curve were generated using the iNEXT package.

The influence of landscape patterns on the diversity of true bugs across different habitat types was evaluated using generalized additive mixed models (GAMMs). Models were developed at seven spatial scales: 250, 500, 1000, 1500, 2000 m, 3000 and 5000 m. Response variables included species richness, abundance, functional richness, functional evenness, and functional divergence. All models were fitted using a Tweedie distribution with a log link function. Sampling team were treated as random factors, and landscape pattern indices at various spatial scales served as explanatory variables. To assess the relationship between species richness and functional diversity, the Pearson correlation coefficient was employed. Multicollinearity among covariates was assessed using the Variance Inflation Factor (VIF), and landscape pattern variables at different spatial scales were sequentially removed based on the highest VIF until all remaining VIFs were less than 5 (see Supplementary Table S4). For each model, model performance was evaluated using the Akaike Information Criterion (AIC) to identify the best-fitting model. Although both PD and MPS are functions of the number of patches and the total landscape area at the landscape level, posing a risk of redundancy between these metrics, models incorporating both indicators were retained when they exhibited superior performance compared to models containing only one of these metrics (refer to Supplementary Table S5 for model details). All analyses were conducted in R version 4.2.3. Functional diversity indices were computed using the dbFD function from the FD package. Multiple comparisons and the Kruskal–Wallis test were performed using the agricolae package. The mgcv package was employed to construct GAMMs. VIF calculations were carried out using the vif function in the car package, and variable correlations were analyzed using the GGally package. Visualization of the results was facilitated using the mgcViz and ggplot2 packages in R 4.4.2.

## 3. Result

A total of 3357 bugs were collected, belonging to 22 families and 174 species. Among these, the highest number of species and individuals was collected in natural forests, with a total of 1226 individuals and 121 species. Planted forests collected 923 individuals and 108 species, while cultivated land collected 439 individuals and 72 species. Complex habitats collected 769 individuals and 107 species (Supplementary Tables S6 and S7). Rarefaction and extrapolation curves of different habitats based on true bug individuals indicate that sampling of true bug communities across the four habitat types was adequate (see Supplementary Figure S1).

### 3.1. True Bug Diversity Distribution in Different Habitat Types

**Figure 2:**
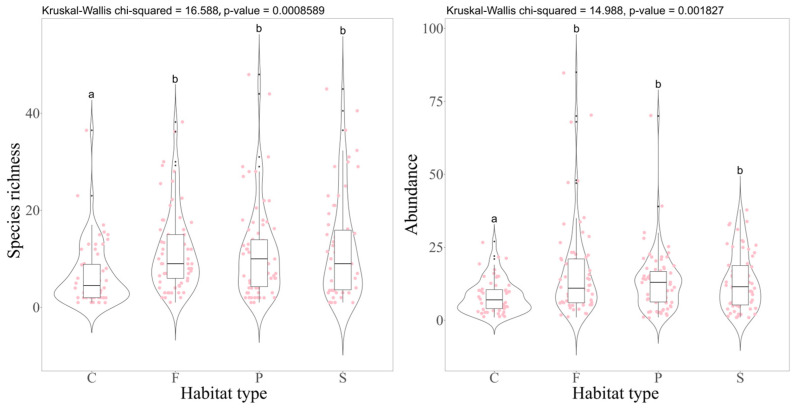
Comparative analysis of true bug diversity across different habitat types. C: cultivated land; F: natural forest; P: planted forest; S: complex habitat. a and b are used to visually indicate whether there are statistically significant differences between different groups. Groups sharing the same letters mean that there is no statistically significant difference in the median between them. Groups with different letters mean that there are statistically significant differences in their medians.

The Kruskal–Wallis test revealed statistically significant differences in species richness (χ^2^ = 16.588, *p* < 0.001) and abundance (χ^2^ = 14.988, *p* < 0.001) of true bugs across various habitat types. Subsequent post hoc comparisons indicated that both the species richness and abundance of true bugs were significantly lower in cultivated lands compared to natural forests, planted forests, and complex habitats (Figure 2). However, indices of functional richness, functional evenness, and functional divergence showed no significant differences among these habitats. Significantly, comparative analyses revealed that functional richness was markedly greater in planted forests compared to cultivated lands (see Supplementary Figure S2).

### 3.2. Response of True Bug Species Richness to Landscape Patterns Across Different Scales in Various Habitats

**Figure 3:**
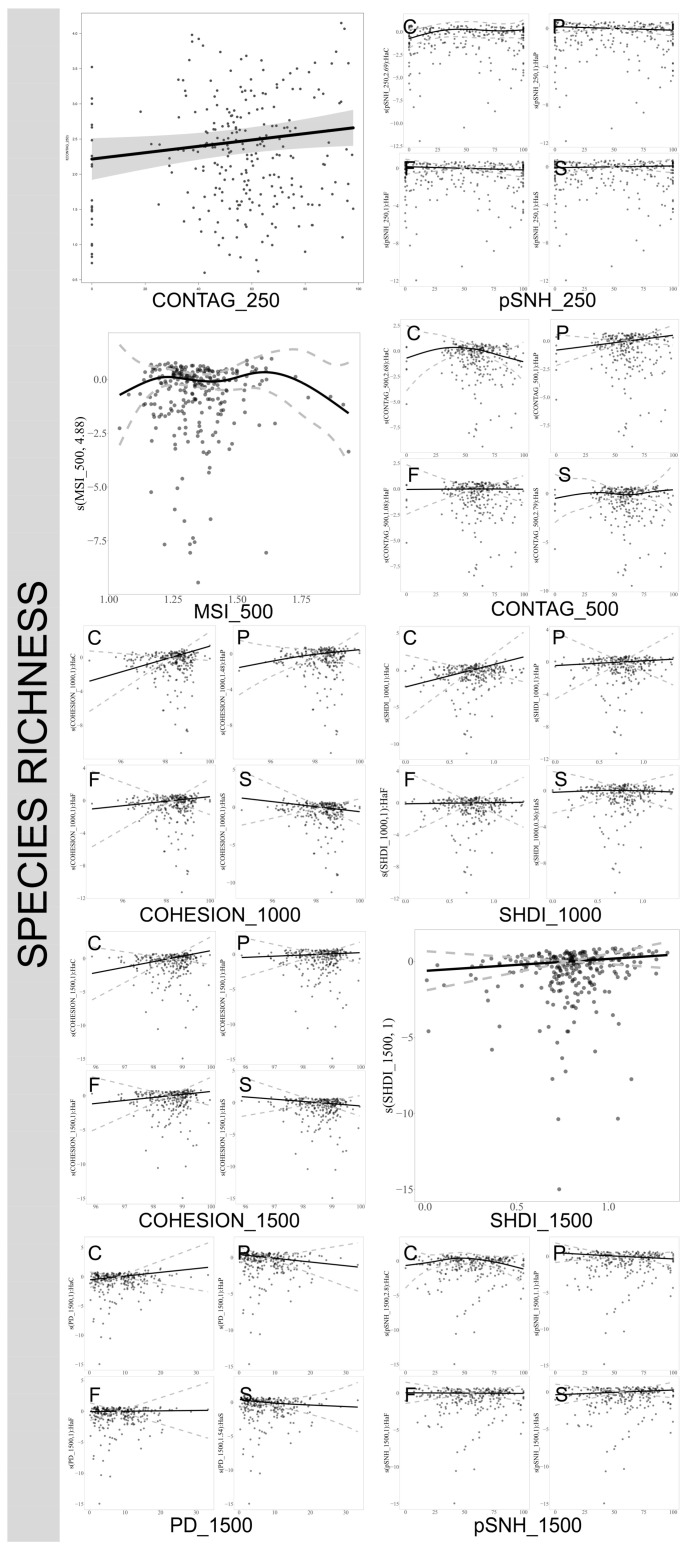
Influence of landscape variables on the richness of true bugs across different spatial scales. Only significant effects are plotted (refer to Supplementary Table S6). C: cultivated lands, F: natural forest, P: planted forest, S: complex habitats. pSNH: proportion of semi-natural habitat, SHDI: Shannon Landscape Diversity Index, PD: Patch Density, MSI: Mean Shape Index, CONTAG: Contagion Index, COHESION: Patch Cohesion Index. The numbers 250, 500, 1000, and 1500 represent the spatial scales of the landscape variables, measured in meters.

The response of true bug species richness to landscape patterns exhibits significant variations across different habitats. At a scale of 250 m, the CONTAG index consistently influences species richness across all habitats, with an increasing trend in richness as the index rises. However, at a scale of 500 m, the impact of the CONTAG index on species richness diminishes in natural forests and complex habitats, while it continues to exert a significant positive effect in planted forests. In cultivated lands, a substantial increase in the CONTAG index paradoxically reduces the species richness of true bugs. The SHDI index impacts true bug species richness positively, but this effect is only evident at larger scales, particularly in cultivated lands. The COHESION index significantly enhances the species richness of true bugs at scales ranging from 1000 m to 5000 m in cultivated lands; a notable positive effect is also observed in planted forests at the 1000 m scale, but this influence weakens with increasing scale. Conversely, an increase in the PD index significantly benefits species richness in cultivated lands, yet exerts a notable negative impact on species richness in planted forests. The pSNH index increase leads to a significant positive effect on species richness in cultivated lands across all studied scales, and a positive influence is detected in complex habitats at scales of 3000 and 5000 m. The MSI index demonstrates that once it reaches a value of approximately 1.6, its influence on the richness of true bug species turns negative. (Figure 3. For plots at 2000 m, 3000 m and 5000 m scales, refer to Supplementary Figure S3).

### 3.3. Response of True Bug Species Abundance to Landscape Patterns Across Different Scales and Habitats

**Figure 4:**
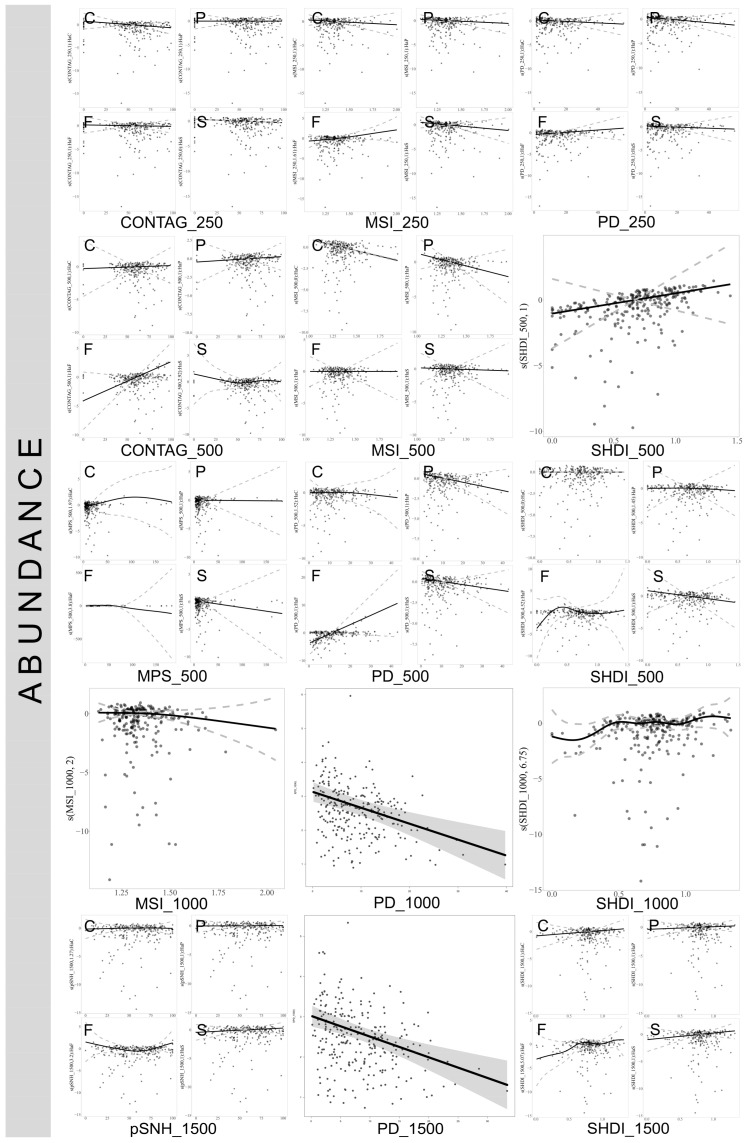
Influence of landscape variables on the abundance of true bugs across different spatial scales. Only significant effects are depicted (refer to Supplementary Table S8). C: cultivated lands, F: natural forest, P: planted forest, S: complex habitats. pSNH: proportion of semi-natural habitat, SHDI: Shannon Landscape Diversity Index, PD: Patch Density, MSI: Mean Shape Index, MPS: Mean Patch Size, CONTAG: Contagion Index. The numbers 250, 500, 1000, and 1500 represent the spatial scales of the landscape variables, measured in meters.

At the 250 m scale, an increase in the CONTAG index significantly negatively impacts the abundance of true bugs in cultivated lands and complex habitats. Conversely, at the 500 m scale, this index exhibits a significant positive effect on true bug abundance in natural forests. Additionally, an increase in the MPS index at the 500 m scale detrimentally affects true bug species abundance in natural forests. At the 3000 m scale, however, an increase in MPS positively influences true bug species abundance in planted forests but negatively impacts it in complex habitats. The increase in the MSI at the 250 m scale positively affects true bug species abundance in natural forests, yet exerts a significant negative effect in complex habitats. At the 500 m scale, an increase in MSI substantially reduces true bug abundance in both cultivated lands and planted forests. The PD index consistently exerts a significant negative impact on true bug abundance across various habitats and scales. Overall, an increase in the SHDI generally has a positive effect on true bug species abundance across different scales. Notably, at the 500 m scale, the influence on species richness in natural forests reaches a peak and then stabilizes. At the 1500 m scale, the positive effects of SHDI on true bug abundance are particularly pronounced in both natural forests and complex habitats. (Figure 4. For plots at 2000 m, 3000 m and 5000 m scales, refer to Supplementary Figure S4).

### 3.4. Response of True Bug Functional Richness to Landscape Pattern Across Different Scales in Varied Habitats

**Figure 5:**
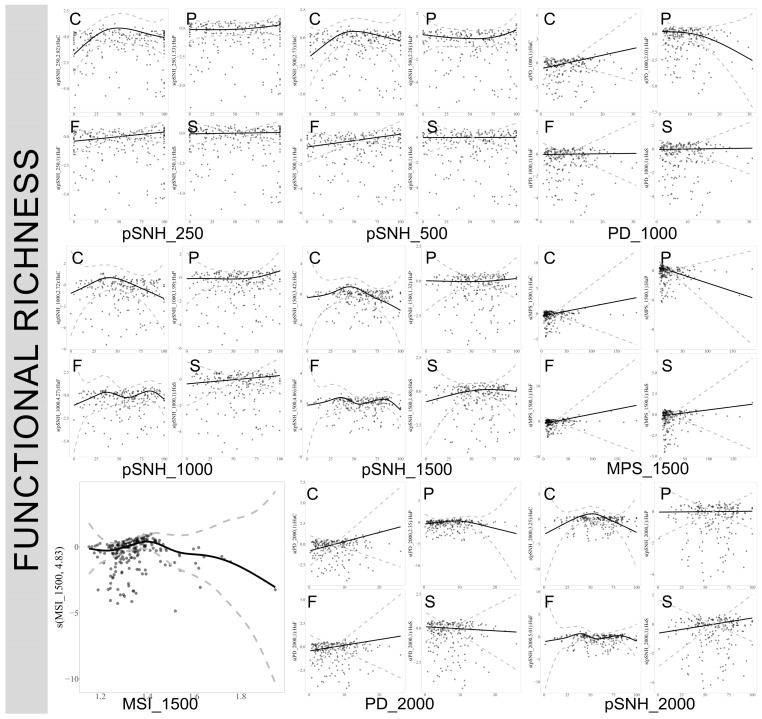
Influence of landscape variables on the functional richness of true bugs across different spatial scales. Only significant impacts are depicted (refer to Supplementary Table S6). C: cultivated lands, F: natural forest, P: planted forest, S: complex habitats. pSNH: proportion of semi-natural habitat, PD: Patch Density, MSI: Mean Shape Index, MPS: Mean Patch Size. The numbers 250, 500, 1000, 1500, and 2000 represent the spatial scales of the landscape variables, measured in meters.

In cultivated lands, the functional richness of true bugs exhibits a significant nonlinear relationship with the proportion of semi-natural habitats at various scales. Specifically, when the proportion of semi-natural habitats in the landscape is below 50%, changes in the pattern positively affect the functional richness of true bugs. However, this positive effect diminishes or becomes negative once the proportion exceeds 50%. Increases in MPS and PD significantly negatively impact the functional richness of true bugs in planted forests at a 500 m scale. Conversely, an increase in MSI generally reduces the functional richness of true bugs across the studied scales. Notably, an increase in PD significantly enhances the functional richness of true bugs in cultivated lands. (Figure 5. For plots at 3000 m and 5000 m scales, refer to Supplementary Figure S5).

### 3.5. Response of True Bug Functional Evenness to Landscape Pattern Scales in Various Habitats

**Figure 6:**
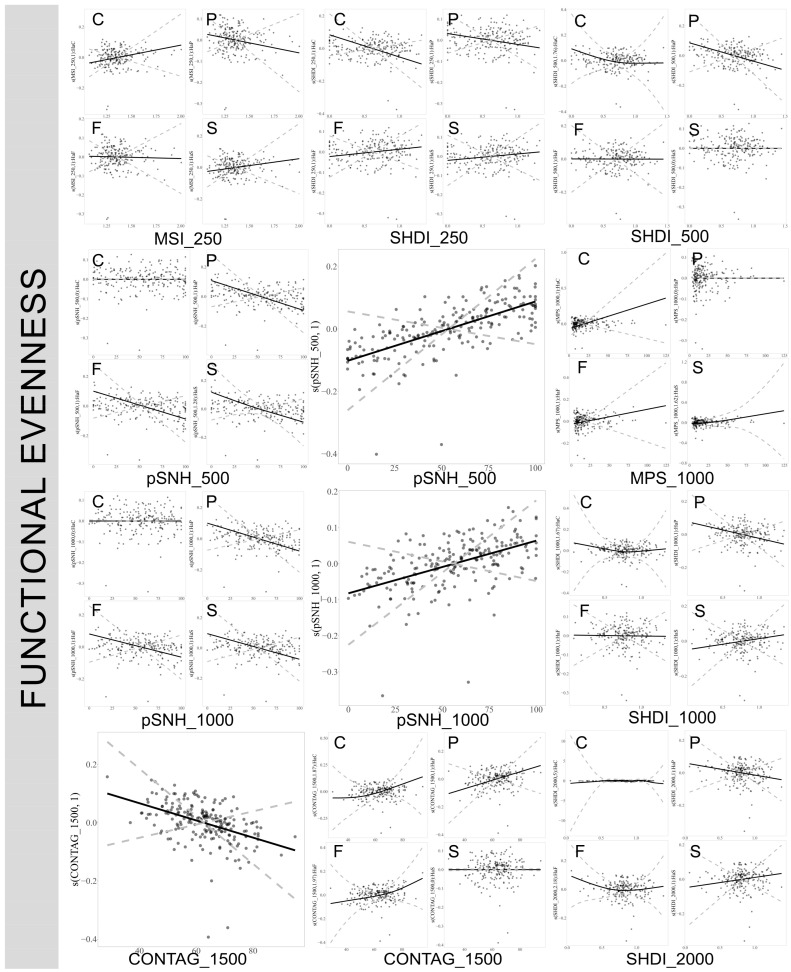
Influence of landscape variables on the functional evenness of true bugs across different spatial scales. Only significant effects are plotted (refer to Supplementary Table S6). C: cultivated lands, F: natural forest, P: planted forest, S: complex habitats. pSNH: proportion of semi-natural habitat, SHDI: Shannon Landscape Diversity Index, MSI: Mean Shape Index, MPS: Mean Patch Size, CONTAG: Contagion Index. The numbers 250, 500, 1000, 1500, and 2000 represent the spatial scales of the landscape variables, measured in meters.

At a 1500 m scale, an increase in the CONTAG index generally exerts a significant negative impact on the functional evenness of true bugs. However, when examining different habitats, this increase in the CONTAG index positively affects the functional evenness of true bugs in both planted forests and natural forests. In contrast, at scales of 500 m and 1000 m, an increased proportion of semi-natural habitats significantly enhances the functional evenness of true bugs. Yet this increase in semi-natural habitats appears to decrease the functional evenness in planted forests, natural forests, and complex habitats. Furthermore, an increase in the SHDI negatively influences the functional evenness of true bugs in planted forests and cultivated lands across various scales. Moreover, increases in the MSI and MPS indices show significant positive effects on true bug functional evenness at scales of 250 m and 1000 m, respectively. (Figure 6. For plots at 3000 m and 5000 m scales, see Supplementary Figure S6).

### 3.6. Response of True Bug Functional Divergence to Landscape Patterns Across Different Scales in Various Habitats

**Figure 7:**
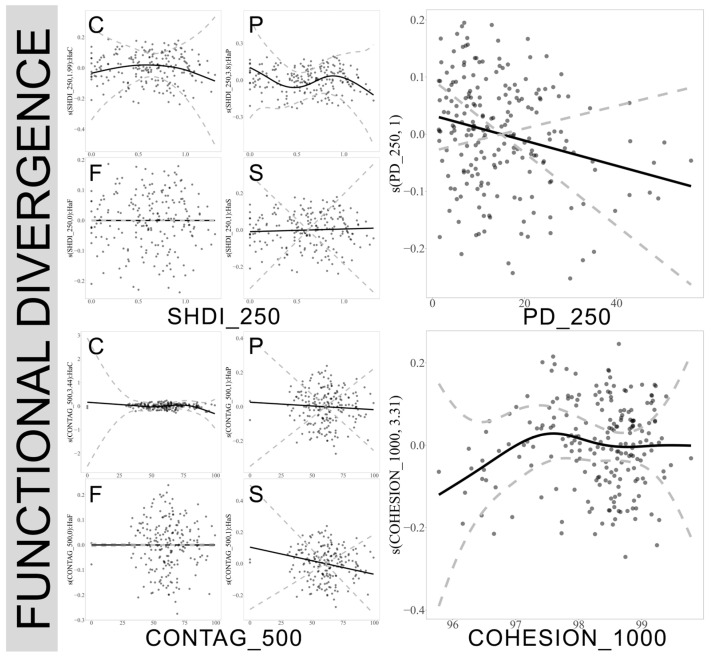
Influence of landscape variables on the functional divergence of true bugs across different spatial scales. Only significant effects are plotted (refer to Supplementary Table S1). C: cultivated lands, F: natural forest, P: planted forest, S: complex habitats. SHDI: Shannon Landscape Diversity Index, PD: Patch Density, CONTAG: Contagion Index, COHESION: Patch Cohesion Index. The numbers 250, 500, and 1000 represent the spatial scales of the landscape variables, measured in meters.

The COHESION index demonstrated a significant nonlinear relationship with the functional divergence of true bugs at a 1000 m scale, where the positive effects diminished when the index value approached approximately 97.5. At larger scales of 3000 m and 5000 m, an increase in the COHESION index generally exerted a significant negative impact on the functional divergence of true bugs. However, this relationship varied across different habitats; an increase in the COHESION index significantly enhanced functional divergence in natural forests, planted forests, and complex habitats, although the positive effect plateaued after reaching a peak in natural forests. At a 3000 m scale, the increase in the MSI index negatively affected the functional divergence of true bugs in planted forests, while it had a significant positive impact in natural forests. At the 250 m scale, an increase in the PD index generally had a negative effect on true bug functional divergence, and a similar negative impact was observed with the increase in the SHDI index in planted forests. (Figure 7. For plots at 3000 m and 5000 m scales, see Supplementary Figure S7).

## 4. Discussion

### 4.1. How Do Differences in Habitat Types Affect the Species Diversity and Functional Diversity of True Bugs?

Habitat modification can alter species composition, resource distribution, and ecological interactions, thereby influencing both taxonomic and functional dimensions of biodiversity [56,57]. In our study, cultivated lands exhibited the lowest species richness and abundance of true bugs, whereas natural forests, planted forests, and complex habitats maintained significantly higher levels. This pattern reflects a combination of factors including superior habitat quality, reduced disturbance, and enhanced landscape connectivity [58,59,60]. Natural forests, characterized by high plant diversity, complex vertical structure, and connectivity, provide abundant resources for foraging, shelter, and reproduction [61,62]. Consequently, greater resource diversity in these habitats favors increased functional divergence and evenness. Planted forests, despite their simpler structure, sustain high true bug diversity, likely due to landscape connectivity. In contrast to the significant variation in taxonomic diversity, functional diversity remained relatively stable across habitats. This suggests that species turnover did not result in major changes in functional structure, likely due to strong functional redundancy within true bug communities. Taxonomically distinct species may perform similar ecological roles, allowing ecosystem functions to be maintained despite shifts in species composition. Functional richness was positively correlated with species richness across habitats, consistent with previous studies [53].

The relatively stable functional diversity across habitats may also reflect the way true bug morphology mediates ecological roles. Traits related to forewing length and hind-femur morphology are associated with dispersal and locomotion, rostrum and fore-femur traits are linked to feeding resource use, body shape reflects habitat use, and eye and antenna traits are related to orientation and sensory ability [35,36,37,38,39,40,41,42,43,44,45,46,47,48,49]. Therefore, even when habitat alteration changes species composition, different true bug species may retain similar combinations of dispersal, feeding, habitat-use, and orientation traits. This trait redundancy or convergence can buffer functional structure against taxonomic turnover, but, if disturbance intensifies or semi-natural habitats are further reduced, functionally similar species may also be lost, potentially weakening ecosystem resilience.

### 4.2. How Does Landscape Structure in Combined Habitats with Different Spatial Scales Influence the Species Diversity and Functional Diversity of True Bugs?

In natural forests, landscape fragmentation at small scales positively correlates with true bug abundance, as fragmented patches act as refuges. However, species richness and functional diversity remain largely unaffected, likely due to dominance by disturbance-tolerant species, which increases functional redundancy [63,64,65]. Patch irregularity and complex shapes at small scales promote abundance, while at larger scales, complex patches enhance species richness by providing more foraging resources and experiencing less disturbance [66,67,68]. High landscape connectivity consistently benefits abundance, species richness, functional evenness, and functional divergence by facilitating dispersal and supporting a broader range of species traits [68,69]. For true bugs, connected landscapes may be particularly important because movement among host plants, refuge sites, and foraging patches depends on dispersal- and orientation-related traits. Additionally, increasing the proportion of semi-natural habitats enhances abundance and functional richness, although functional evenness may decrease, reflecting the role of these habitats as sources of species and trait diversity [70,71].

In planted forests, true bug diversity is generally reduced by higher fragmentation, complex patch shapes, and diverse patch types due to homogeneous vegetation and limited ecological niches [72,73]. Nevertheless, increasing landscape connectivity promotes species richness, abundance, and functional diversity, providing opportunities for the establishment of more species pools [74]. Enhancing understory vegetation and increasing the proportion of semi-natural habitats further improve community diversity, increasing both species richness and functional richness.

In cultivated lands, small, diverse patches—common in intercropping systems—enhance species richness, abundance, functional richness, and functional evenness by providing a variety of ecological niches [75]. Patch type diversity generally increases species richness but may reduce functional evenness, while irregular patch shapes at small scales reduce abundance but increase functional evenness. Connectivity of dominant patch types at small scales can reduce abundance and species richness but enhances functional divergence through a rescue effect for functional traits [76]. Increasing the proportion of semi-natural habitats also promotes species richness and functional richness, with functional richness reaching a maximum at approximately 50% coverage, though functional evenness may decrease [77,78,79,80].

In complex habitats, high fragmentation in the surrounding landscape increases species richness and abundance at smaller scales but can reduce abundance at larger scales, likely reflecting a balance between resource availability and disturbance intensity. Increased diversity of patch types promotes abundance at larger scales, while greater patch shape complexity reduces abundance at smaller scales but enhances species richness at larger scales, likely due to the provision of additional foraging resources and reduced disturbance in irregular patches [66,67,68]. Enhancing landscape connectivity at larger scales improves species richness, functional divergence, and functional evenness, suggesting that connected landscapes facilitate dispersal and support more complementary functional traits [68,69]. Similarly, increasing the proportion of semi-natural habitats benefits species richness and functional richness, although functional evenness may decline, indicating that semi-natural areas serve as sources of diverse traits but may reduce complementarity within the functional trait space [70,71,77,79].

## 5. Conclusions

Establishing priority areas for biodiversity conservation is crucial for maintaining biodiversity. However, balancing conservation goals with economic development remains a significant challenge. Beyond establishing national nature reserves that exclude human activity, maintaining biodiversity within agricultural landscapes has become an urgent issue. This study, grounded in priority areas for biodiversity conservation, explores the influence of habitat characteristics and landscape patterns on true bug diversity across spatial scales. The goal is to propose context-specific biodiversity conservation strategies based on varying habitat conditions, avoiding a one-size-fits-all approach.

We emphasize that the impact of landscape patterns on true bug community diversity exhibits inconsistent trends across different habitats. Regardless of patch size, natural forests are worthy of conservation. Furthermore, enhancing landscape connectivity and reducing human disturbance can further benefit the conservation of bug diversity. For planted forests, where changing patch size and disturbance levels may not be feasible, increasing landscape connectivity can effectively improve true bug diversity protection. Furthermore, in cultivated lands, the reality of individually owned small-scale farms presents immutable constraints. For agricultural areas, the idea of substantially increasing the proportion of semi-natural habitats in the landscape is impractical. Our findings indicate that intercropping various plant species and enhancing the diversity of patch types within the landscape promotes true bug diversity. Additionally, increasing natural vegetation along field margins and roadsides (reducing the cementation of secondary farm roads to foster natural vegetation growth) enhances landscape connectivity and the proportion of semi-natural vegetation (with a target area ratio increase to 46–50% in feasible regions), thereby more effectively safeguarding true bug diversity. However, while herbivorous true bugs are a headache for farmers as agricultural pests, predatory true bugs are excellent ecological pest control helpers in agricultural areas. Their conservation and management in agricultural areas still need to be further explored according to their different feeding habits. Complex habitats present a more complex scenario where, despite the immutable nature of agricultural land, increasing landscape connectivity and maintaining a higher proportion of semi-natural habitats can help protect true bug diversity. In summary, it is imperative to recognize that influencing factors do not operate in isolation but should be considered in terms of their synergistic interactions.

Temporal variation associated with seasonal changes and potential elevational effects may have influenced community composition and diversity patterns. Although all habitats were sampled within the same regional landscape context, the observed differences among habitat types may therefore partially reflect temporal or elevational variation. Future studies incorporating repeated seasonal sampling and explicitly accounting for elevation as a covariate would help disentangle habitat effects from temporal and environmental influences.

## Data Availability

Traits data are available from a publicly repository (https://doi.org/10.6084/m9.figshare.25376458.v1), or will become publicly available after an embargo period of five years from the end of data assembly to give the owners and collectors of the data time to perform their analysis. Any other relevant data are available from the corresponding author upon reasonable request.

## Supplementary Materials

The following supporting information can be downloaded at: https://www.mdpi.com/article/10.3390/insects17050497/s1.
