# Supplementary material for: The Joint Effects of Habitat Types and Surrounding Landscape Patterns on the Diversity of True Bugs in Southwest China
Source: Insects. 2026 May 13;17(5):497. doi: 10.3390/insects17050497 (PMC13207312; doi:10.3390/insects17050497)
Supplement: Supplementary file 1 [file insects-17-00497-s001.zip › insects-4260817-supplementary.pdf]

Supplementary Information:

The joint effects of habitat types and surrounding landscape patterns on the diversity of true bugs in Southwest China

Shutong Gao[1, 2], Zhixing Lu[1, 3], Xiang Zhang[1, 3], Qiao Li[4] & Youqing Chen[1, 3]✉

✉Corresponding author: cyqcaf@126.com

Table S1. Overview of morphological functional traits calculated from the measured morphological characteristics (Gossner et al., 2015).

Trait category	Morphometric trait	Type	Description
Body size	Body length	numeric	Total length
Dispersal ability	Rel. Forewing length	numeric	Forewing length / body length
	Hind-Femur shape	numeric	Femur length / femur width
	Rel. Hind-Femur length	numeric	Femur length / body length
Feeding resource use	Rel. Rostrum length	numeric	Rostrum length / body length
	Front-Femur shape	numeric	Femur length / femur width
Habitat use	Body shape	numeric	Body length / body width
Orientation	Rel. Eye size	numeric	Eye width / head width
	Rel. Antenna length	numeric	Total antenna length /body length

Table S2. Results of normality testing using the Shapiro-Wilk statistical method. The same applies to subsequent analyses. FRic: functional richness; FEve: functional evenness; FDiv: functional divergence.

	W	df	p-value
Species richness	0.852	260	0
Abundance	0.502	260	0
FRic	0.83	210	0
FEve	0.907	210	0
FDiv	0.989	210	0.091

Note: P > 0.05 indicates conformity with a normal distribution.

Table S3. Homogeneity of variance test based on the mean using Levene's test.

	F	df1	df2	p-value
Species richness	3.733	3	256	0.012
Abundance	5.086	3	256	0.002
FRic	1.909	3	206	0.129
FEve	0.59	3	206	0.622
FDiv	1.584	3	206	0.194

Note: P < 0.05 indicates heteroscedasticity.

Table S4. Variables included in the initial model and their VIF values after exclusion of variables with high VIFs. NA indicates that the variable was excluded from the initial model at the corresponding spatial scale.

Sacle	PD	MPS	MSI	CONTAG	COHESION	SHDI	pSNH
250m	2.172319	NA	1.496665	1.157272	NA	2.264389	1.164454
500m	2.186587	3.126529	1.352511	3.214924	NA	3.832327	1.285176
1000m	3.523429	2.399131	1.741666	3.420563	4.198102	3.762089	1.255693
1500m	3.170195	2.737562	2.163982	3.482519	3.859131	3.427826	1.232038
2000m	3.543723	3.388116	2.735232	3.659257	4.242001	3.824529	1.181782
3000m	3.31542	3.814191	3.194155	3.511083	3.631911	4.128475	1.192294

5000m	3.621143	NA	1.19631	3.201051	2.123217	NA	1.247011

Table S5. Generalized additive mixed-effects models established for each spatial scale, with the best model selected based on Akaike Information Criterion (AIC) values. The numbers 250, 500, 1000, 1500, and 2000 denote the spatial scales of landscape variables, measured in meters. Ha: habitat type; T: sampling team.

Variable	Scale	Model	df	AIC	R-sq.(adj)	Deviance explained	-REML
Richness	250m	~Ha+s(pSNH_250,by=Ha)+s(PD_250)+CONTAG_250+s(T,bs='re')	15.27766	1690.044	0.122	21.60%	852.95
Richness	500m	~Ha+s(CONTAG_500,by=Ha)+s(MSI_500)+s(pSNH_500)+s(T,bs='re')	22.88026	1677.669	0.189	29%	845.44
Richness	1000m	~Ha+s(MPS_1000,by=Ha)+s(COHESION_1000,by=Ha)+s(SHDI_1000,by=Ha)+s(SHDI_1000)+s(T,bs='re')	29.70634	1662.548	0.19	36.10%	836.73
Richness	1500m	~Ha+s(PD_1500,by=Ha)+s(COHESION_1500,by=Ha)+s(pSNH_1500,by=Ha)+s(SHDI_1500)+s(T,bs='re')	23.61994	1680.849	0.158	28.60%	845.17
Richness	2000m	~Ha+s(COHESION_2000,by=Ha)+s(SHDI_2000,by=Ha)+MPS_2000+s(T,bs='re')	21.70262	1678.583	0.175	28.20%	846.15
Richness	3000m	~Ha+s(MPS_3000,by=Ha)+s(COHESION_3000,by=Ha)+s(pSNH_3000,by=Ha)+s(T,bs='re')	35.65317	1658.081	0.253	39.80%	836.81
Richness	5000m	~Ha+s(PD_5000,by=Ha)+s(MSI_5000,by=Ha)+s(COHESION_5000,by=Ha)+s(pSNH_5000,by=Ha)+s(T,bs='re'),data=gts,method="REML"	40.40814	1656.715	0.325	42.20%	837.39
Abundance	250m	~Ha+s(PD_250,by=Ha)+s(MSI_250,by=Ha)+s(CONTAG_250,by=Ha)+s(SHDI_250,by=Ha)+s(CONTAG_250)+s(SH	27.93599	1817.154	0.142	28.30%	912.31

		DI_250)+s(T,bs='re')					
Abundance	500m	~Ha+s(PD_500,by=Ha,k=7)+s(MPS_500,by=Ha,k=7)+s(MSI_500,by=Ha,k=7)+s(CONTAG_500,by=Ha,k=7)+s(SHDI_500,by=Ha,k=7)+s(pSNH_500,by=Ha,k=7)+s(MSI_500,k=7)+s(SHDI_500,k=7)+s(T,bs='re')	52.73326	1794.074	0.169	45.40%	912.72
Abundance	1000m	~Ha+s(pSNH_1000,by=Ha)+PD_1000+s(MSI_1000)+s(SHDI_1000)+s(T,bs='re')	26.00516	1801.533	0.0813	33.40%	909.4
Abundance	1500m	~Ha+s(SHDI_1500,by=Ha)+s(pSNH_1500,by=Ha)+PD_1500+s(MSI_1500)+s(T,bs='re')	25.65620	1815.595	0.0979	27.40%	916.93
Abundance	2000m	~Ha+s(PD_2000,by=Ha)+s(MPS_2000,by=Ha)+s(MSI_2000)+s(SHDI_2000)+s(T,bs='re')	25.53886	1809.5	0.0742	28.90%	913.77
Abundance	3000m	~Ha+s(MPS_3000,by=Ha)+s(COHESION_3000,by=Ha)+s(COHESION_3000)+s(SHDI_3000)+s(T,bs='re')	31.27312	1809.946	0.103	31.70%	911.03
Abundance	5000m	~Ha+s(PD_5000,by=Ha)+s(COHESION_5000)+s(pSNH_5000)+s(T,bs='re')	23.40480	1802.497	0.109	29.50%	907.85
FRic	250m	~Ha+s(pSNH_250,by=Ha)+s(T,bs='re')	14.44767	-1294.302	0.1	16.60%	-647.72
FRic	500m	~Ha+s(pSNH_500,by=Ha)+s(MPS_500)+s(MSI_500)+s(CONTAG_500)+s(T,bs='re')	20.11974	-1285.331	0.112	17.50%	-641.49
FRic	1000m	~Ha+s(PD_1000,by=Ha)+s(pSNH_1000,by=Ha)+s(MSI_1000)+s(T,bs='re')	27.66752	-1287.732	0.15	23.40%	-643.06

FRic	1500m	~Ha+s(PD_1500,by=Ha)+s(MPS_1500,by=Ha)+s(pSNH_1500,by=Ha)+s(PD_1500)+s(MSI_1500)+s(COHESION_1500)+s(T,bs='re')	39.14746	-1302.633	0.193	35%	-648.41
FRic	2000m	~Ha+s(PD_2000,by=Ha)+s(SHDI_2000,by=Ha)+s(pSNH_2000,by=Ha)+s(T,bs='re')	33.53870	-1296.559	0.209	30%	-647.84
FRic	3000m	~Ha+s(SHDI_3000,by=Ha)+s(T,bs='re')	14.92434	-1287.449	0.122	14.60%	-644.92
FRic	5000m	~Ha+s(COHESION_5000,by=Ha)+s(MSI_5000)+s(pSNH_5000)+s(T,bs='re')	21.23915	-1279.84	0.113	16.40%	-639.04
FEve	250m	~Ha+s(MSI_250,by=Ha)+s(SHDI_250,by=Ha)+s(pSNH_250,by=Ha)+s(pSNH_250)+s(T,bs='re')	22.92631	-555.441	0.123	20.50%	-213.06
FEve	500m	~Ha+s(PD_500,by=Ha)+s(SHDI_500,by=Ha)+s(pSNH_500,by=Ha)+s(PD_500)+s(SHDI_500)+s(pSNH_500)+s(T,bs='re')	19.88591	-540.3968	0.0439	12%	-223.06
FEve	1000m	~Ha+s(MPS_1000,by=Ha)+s(SHDI_1000,by=Ha)+s(pSNH_1000,by=Ha)+s(MPS_1000)+s(CONTAG_1000)+s(pSNH_1000)+s(T,bs='re')	23.29241	-544.6342	0.0791	16.60%	-222.85
FEve	1500m	~Ha+s(CONTAG_1500,by=Ha)+s(MPS_1500)+s(CONTAG_1500)+s(T,bs='re')	12.84681	-544.2643	0.031	7.66%	-246.1
FEve	2000m	~Ha+s(SHDI_2000,by=Ha)+s(MPS_2000)+s(CONTAG_2000)+s(T,bs='re')	17.94814	-560.0555	0.123	18.40%	-247.99
FEve	3000m	~Ha+s(CONTAG_3000)+s(SHDI_3000)+s(T,bs='re')	8.755555	-543.7225	0.00827	3.74%	-255.54

FEve	5000m	~Ha+s(PD_5000,by=Ha)+s(COHESION_5000,by=Ha)+s(MSI_5000)+s(T,bs='re')	26.96436	-548.792	0.118	21%	-235.31
FDiv	250m	~Ha+s(CONTAG_250,by=Ha)+s(SHDI_250,by=Ha)+s(PD_250)+s(CONTAG_250)+s(SHDI_250)+s(T,bs='re')	24.01262	-356.6787	0.123	20.20%	-145.58
FDiv	500m	~Ha+s(CONTAG_500,by=Ha)+s(CONTAG_500)+s(T,bs='re')	12.07884	-350.0354	0.0341	7.77%	-155.05
FDiv	1000m	~s(COHESION_1000)+s(T,bs='re')	6.167278	-357.8363	0.0447	5.99%	-173.58
FDiv	1500m	~Ha+s(PD_1500,by=Ha,k=3)+s(MPS_1500,by=Ha,k=3)+s(MSI_1500,by=Ha,k=3)+s(CONTAG_1500,by=Ha,k=3)+s(COHESION_1500,by=Ha,k=3)+s(SHDI_1500,by=Ha,k=3)+s(pSNH_1500,by=Ha,k=3)+s(PD_1500,k=3)+s(MPS_1500,k=3)+s(MSI_1500,k=3)+s(CONTAG_1500,k=3)+s(COHESION_1500,k=3)+s(SHDI_1500,k=3)+s(pSNH_1500,k=3)+s(T,bs='re')	36.29577	-321.501	0.00122	16.10%	-87.036
FDiv	2000m	~Ha+s(PD_2000,by=Ha,k=3)+s(MPS_2000,by=Ha,k=3)+s(MSI_2000,by=Ha,k=3)+s(CONTAG_2000,by=Ha,k=3)+s(COHESION_2000,by=Ha,k=3)+s(SHDI_2000,by=Ha,k=3)+s(pSNH_2000,by=Ha,k=3)+s(PD_2000,k=3)+s(MPS_2000,k=3)+s(MSI_2000,k=3)+s(CONTAG_2000,k=3)+s(COHESION_2000,k=3)+s(SHDI_2000,k=3)	39.16926	-328.5003	0.0481	21%	-90.746

		+s(pSNH_2000,k=3)+s(T,bs='re')					
FDiv	3000m	~Ha+s(MPS_3000,by=Ha)+s(MSI_3000,by=Ha)+s(COHESION_3000,by=Ha)+s(MPS_3000)+s(MSI_3000)+s(CONTAG_3000)+s(COHESION_3000,k=3)+s(pSNH_3000)+s(T,bs='re')	30.29688	-353.3348	0.132	23.70%	-131.78
FDiv	5000m	~Ha+s(COHESION_5000,by=Ha)+s(COHESION_5000)+s(T,bs='re')	179728	-351.9407	0.0495	9.56%	-155.96

Table S6. composition and structure of Heteroptera in the Xishuangbanna Priority Areas of Biodiversity Conservation

**Superfamily**	**Family**	**Genera**		**Species**		**individuals**	
		**number**	**proportion（%）**	**number**	**proportion（%）**	**number**	**proportion（%）**
Pentatomoidea	Acanthosomatidae	1	0.91	1	0.57	4	0.12
	Cydnidae	1	0.91	2	1.15	6	0.18
	inidoridae	3	2.73	7	4.02	22	0.66
	Pentatomidae	21	19.09	32	18.39	831	24.75
	lataspidae	3	2.73	8	4.60	430	12.81
	Scutelleridae	6	5.45	6	3.45	30	0.89
	Tessaratomidae	5	4.55	6	3.45	41	1.22
	Urostylididae	2	1.82	3	1.72	3	0.09
Pyrrhocoroidea	Largidae	2	1.82	3	1.72	17	0.51
	Pyrrhocoridae	4	3.64	6	3.45	92	2.74
Coreoidea	Alydidae	2	1.82	5	2.87	2	0.06
	Coreidae	22	20.00	47	27.01	136	4.05
	Rhopalidae	1	0.91	1	0.57	128	3.81
Lygaeoidea	Berytidae	2	1.82	2	1.15	173	5.15
	Blissidae	2	1.82	2	1.15	666	19.84
	Geocoridae	1	0.91	3	1.72	6	0.18
	Lygaeidae	3	2.73	3	1.72	63	1.88
	Malcidae	1	0.91	2	1.15	14	0.42
	Rhyparochromidae	11	10.00	15	8.62	30	0.89
Naboidea	Nabidae	2	1.82	2	1.15	176	5.24

Superfamily	Family	Genera		Species		individuals	
		number	proportion（%）	number	proportion（%）	number	proportion（%）
Reduvioidea	Reduviidae	12	10.91	15	8.62	43	1.28
Miroidea	Miridae	3	2.73	3	1.72	444	13.23
Total		110		174		3357	

Table S7. Composition of Heteroptera in different habitat types

Habitat type	Family		Genera		Species		individuals	
	number	proportion（%）	number	proportion（%）	number	proportion（%）	number	proportion（%）
Cultivated land	16	72.73	51	46.36	72	41.38	439	13.08
Natural forest	22	100.00	81	73.64	121	69.54	1226	36.52
Planted forest	19	86.36	70	63.64	108	62.07	923	27.49
Complex habitat	20	90.91	76	69.09	107	61.49	769	22.91
Total	22		110		174		3357	

Table S8. Optimal fitting results for the best generalized additive mixed model, listing only statistically significant terms. The numbers 250, 500, 1000, 1500, and 2000 denote the spatial scales of the landscape variables, measured in meters. HaC: cultivated land; HaF: natural forest; HaP: planted forest; HaS: complex habitat.

Variable 1	Variable 2	edf	Ref.df	t / F value	Estimate	k-index	p-value
Richness	pSNH_250:HaC	2.6868	3.277	4.192		0.99	0.01
Richness	CONTAG_250			2.076	0.004528		0.04
Richness	CONTAG_500:HaC	2.6794	3.341	4.63		0.92	<0.01
Richness	CONTAG_500:HaP	1.0002	1	8.625		0.92	<0.01
Richness	MSI_500	4.8754	5.972	3.275		1.07	<0.01
Richness	COHESION_1000:HaC	1.0002	1.0005	15.371		0.89	<0.01
Richness	COHESION_1000:HaP	1.4845	1.8311	4.427		0.89	0.05
Richness	SHDI_1000:HaC	1.0001	1.0003	7.024		0.95	0.01
Richness	SHDI_1000	5.9782	7.1033	3.462		0.95	<0.01
Richness	PD_1500:HaC	1.0002	1	3.72		0.89	0.05
Richness	PD_1500:HaP	1.0003	1.001	3.834		0.89	0.05
Richness	COHESION_1500:HaC	1.0002	1	8		0.98	0.01
Richness	pSNH_1500:HaC	2.8014	3.502	2.968		1.03	0.03
Richness	SHDI_1500	1.0006	1.001	5.777		0.93	0.02
Richness	COHESION_2000:HaC	1.305	1.558	12.493		0.9	<0.01
Richness	SHDI_2000:HaC	1.0002	1	18.858		1.08	<0.01

Richness	MPS_3000:HaP	3.256	3.802	4.801		1.05	<0.01
Richness	MPS_3000:HaS	2.5746	3.144	4.592		1.05	<0.01
Richness	COHESION_3000:HaC	3.0749	3.85	3.643		0.91	0.01
Richness	COHESION_3000:HaS	1.0229	1.045	14.374		0.91	<0.01
Richness	pSNH_3000:HaC	3.9799	4.887	2.797		1.04	0.02
Richness	pSNH_3000:HaS	1.0001	1	5.717		1.04	0.02
Richness	PD_5000:HaP	1	1	14.778		0.93	<0.01
Richness	MSI_5000:HaS	3.185	3.936	4.685		0.92	<0.01
Richness	COHESION_5000:HaC	1	1	6.237		0.94	0.01
Richness	COHESION_5000:HaS	3.984	4.868	4.105		0.94	<0.01
Richness	pSNH_5000:HaS	1	1	7.564		1.01	0.01
Abundance	PD_250:HaP	1.0001404	1.0002787	5.817		0.9	0.02
Abundance	PD_250:HaF	1.0003315	1.0006614	3.729		0.9	0.05
Abundance	MSI_250:HaF	1.6149429	2.019416	8.182		0.94	<0.01
Abundance	MSI_250:HaS	1.0001219	1.0002429	4.824		0.94	0.03
Abundance	CONTAG_250:HaC	1.0000751	1.000146	5.434		0.93	0.02
Abundance	CONTAG_250:HaS	0.0001456	0.0002843	7456000		0.93	<0.01
Abundance	PD_500:HaF	1.0000046	1.0000084	19.54		0.91	<0.01
Abundance	PD_500:HaP	1.0003338	1.0006631	8.393		0.91	<0.01
Abundance	PD_500:HaS	1.0003688	1.000728	4.981		0.91	0.03
Abundance	MPS_500:HaF	3.8013775	3.9795171	4.998		0.92	<0.01
Abundance	MSI_500:HaC	0.0002268	0.00044	11330000		1.07	<0.01
Abundance	MSI_500:HaP	1.0002276	1.0004405	7.083		0.98	0.01
Abundance	CONTAG_500:HaF	1.0006976	1.0013497	16.92		0.98	<0.01
Abundance	SHDI_500:HaF	4.5226644	5.2481901	6.91		0.98	<0.01
Abundance	pSNH_500:HaF	5.2027094	5.7193805	4.203		1.04	<0.01
Abundance	SHDI_500	1.0003131	1.0005675	3.855			0.05
Abundance	PD_1000			-4.299	-0.04704		<0.01
Abundance	pSNH_1000:HaF	3.3867	4.175	3.067		0.89	0.01
Abundance	MSI_1000	2.0031	2.566	3.326		0.93	0.03
Abundance	SHDI_1000	6.7493	7.854	5.244		0.98	<0.01
Abundance	PD_1500			-4.312	-0.05141		<0.01
Abundance	SHDI_1500:HaF	5.0724	6.18	3.206		0.92	<0.01
Abundance	SHDI_1500:HaS	1.0002	1	4.832		0.92	0.03
Abundance	pSNH_1500:HaF	3.1965	3.982	3.225		0.97	0.01
Abundance	PD_2000:HaC	1.0003	1.001	3.979		1	0.05
Abundance	PD_2000:HaP	1.0001	1	7.091		1	0.01
Abundance	SHDI_2000	1.8977	2.418	3.331		1.13	0.02
Abundance	MPS_3000:HaP	1.0000644	1.0001274	7.267		1.04	0.01
Abundance	MPS_3000:HaS	2.4458776	2.9870175	3.653		1.04	0.01
Abundance	COHESION_3000:HaC	0.0003153	0.0006082	4073000		0.91	<0.01
Abundance	COHESION_3000:HaF	5.3930677	6.3673721	2.788		0.91	0.01
Abundance	COHESION_3000	3.0596515	3.88006	2.926		0.91	0.02
Abundance	PD_5000:HaC	1.0002	1	12.291		0.93	<0.01

Abundance	PD_5000:HaF	4.555	5.315	2.487	0.93	0.04
Abundance	PD_5000:HaP	1.0001	1	17.528	0.93	<0.01
FRic	pSNH_250:HaC	2.9164	3.583	6.681	0.96	<0.01
FRic	pSNH_250:HaF	1.0005	1.001	4.18	0.94	0.04
FRic	pSNH_500:HaC	2.735	3.403	3.914	0.98	0.01
FRicc	pSNH_500:HaF	1	1	5.138	0.98	0.02
FRic	PD_1000:HaC	1.0001	1	4.875	0.92	0.03
FRic	PD_1000:HaP	2.0335	2.569	3.488	0.92	0.02
FRic	pSNH_1000:HaC	2.7186	3.36	3.277	0.92	0.02
FRic	MPS_1500:HaP	1.0001	1	8.341	0.97	<0.01
FRic	pSNH_1500:HaC	3.4202	4.224	3.744	0.95	<0.01
FRic	pSNH_1500:HaF	4.855	5.811	2.26	0.95	0.04
FRic	MSI_1500	4.8345	5.918	3.701	0.96	<0.01
FRic	PD_2000:HaC	1.0001	1	4.668	0.91	0.03
FRic	pSNH_2000:HaC	3.2453	4.063	3.569	0.99	0.01
FRic	SHDI_3000:HaP	2.7323	3.436	3.069	0.91	0.02
FRic	COHESION_5000:HaP	3.7522	4.603	2.841	0.98	0.02
FEve	MSI_250:HaC	1	1	3.944	0.98	0.05
FEve	SHDI_250:HaC	1	1	10.646	0.99	<0.01
FEve	SHDI_500:HaP	1	1	4.837	0.97	0.03
FEve	pSNH_500:HaF	1	1	7.662	0.97	0.01
FEve	pSNH_500:HaP	1	1	9.391	0.97	<0.01
FEve	pSNH_500:HaS	1.279	1.517	6.483	0.97	0.01
FEve	pSNH_500	1	1	10.375	0.97	<0.01
FEve	MPS_1000:HaC	1	1	8.23	1.05	<0.01
FEve	SHDI_1000:HaP	1	1	4.466	1.06	0.04
FEve	pSNH_1000:HaF	1	1	5.303	1.02	0.02
FEve	pSNH_1000:HaP	1	1	8.342	1.02	<0.01
FEve	pSNH_1000:HaS	1	1	6.599	1.02	0.01
FEve	pSNH_1000	1	1	8.218	1.02	<0.01
FEve	CONTAG_1500:HaF	1.971	2.4975086	3.124	1.07	0.05
FEve	CONTAG_1500:HaP	1	1.000008	5.876	1.07	0.02
FEve	CONTAG_1500	1	1.00009	7.999	1.07	0.01
FEve	SHDI_2000:HaC	4.9987	5.577	4.026	1.09	0.01
FEve	COHESION_5000:HaS	5.454	6.469	2.901	1.04	0.01
FDiv	SHDI_250:HaP	3.802	4.668	2.923	0.99	0.03
FDiv	PD_250	1	1	6.948	0.97	0.01
FDiv	CONTAG_500:HaC	3.438	4.2351104	2.402	1.05	0.05
FDiv	COHESION_1000	3.3059469	4.167	2.482	1.04	0.04
FDiv	MPS_3000:HaF	1.199	1.261	4.346	1	0.02
FDiv	MSI_3000:HaP	1	1	4.934	1.09	0.03
FDiv	COHESION_3000:HaF	2.66	3.314	2.603	1.03	0.04
FDiv	COHESION_5000:HaP	2.946	3.832	3.724	1.11	0.02
FDiv	COHESION_5000:HaS	1	1	4.026	1.11	0.05

FDiv	COHESION_5000	1	1	3.984	1.11	0.05

Table S9. Pearson correlation between elevation and landscape variables

Variable 1	Variable 2	r	ρ 的 95% 置信区间	P
PD_250	Ele	-0.128	(-0.246, -0.007)	0.039
PD_500	Ele	-0.177	(-0.292, -0.056)	0.004
PD_1000	Ele	-0.238	(-0.350, -0.120)	0
PD_1500	Ele	-0.238	(-0.349, -0.119)	0
PD_2000	Ele	-0.238	(-0.349, -0.119)	0
MPS_250	Ele	-0.089	(-0.208, 0.033)	0.154
AREA_MN_500	Ele	0.024	(-0.098, 0.146)	0.696
AREA_MN_1000	Ele	0.064	(-0.058, 0.184)	0.302
AREA_MN_1500	Ele	0.092	(-0.030, 0.211)	0.14
AREA_MN_2000	Ele	0.118	(-0.004, 0.236)	0.058
MSI_250	Ele	0.074	(-0.048, 0.194)	0.232
SHAPE_MN_500	Ele	0.127	(0.006, 0.245)	0.04
SHAPE_MN_1000	Ele	0.038	(-0.084, 0.159)	0.539
SHAPE_MN_1500	Ele	-0.003	(-0.125, 0.119)	0.959
SHAPE_MN_2000	Ele	0.038	(-0.084, 0.159)	0.546
CONTAG_250	Ele	0.122	(0.000, 0.240)	0.049
CONTAG_500	Ele	-0.081	(-0.200, 0.041)	0.194
CONTAG_1000	Ele	-0.037	(-0.158, 0.085)	0.547
CONTAG_1500	Ele	-0.008	(-0.130, 0.113)	0.894
CONTAG_2000	Ele	0.026	(-0.096, 0.147)	0.677
COHESION_250	Ele	0.021	(-0.101, 0.142)	0.735
COHESION_500	Ele	0.137	(0.015, 0.254)	0.027
COHESION_1000	Ele	0.272	(0.155, 0.381)	0
COHESION_1500	Ele	0.281	(0.165, 0.389)	0
COHESION_2000	Ele	0.254	(0.137, 0.365)	0
SHDI_250	Ele	0.03	(-0.092, 0.151)	0.626
SHDI_500	Ele	-0.001	(-0.123, 0.121)	0.986
SHDI_1000	Ele	-0.067	(-0.187, 0.055)	0.283
SHDI_1500	Ele	-0.093	(-0.212, 0.029)	0.134
SHDI_2000	Ele	-0.126	(-0.244, -0.005)	0.042
pSNH250	Ele	0.165	(0.045, 0.281)	0.008
pSNH500	Ele	0.196	(0.076, 0.310)	0.002
pSNH1000	Ele	0.179	(0.058, 0.294)	0.004
pSNH1500	Ele	0.142	(0.021, 0.259)	0.022
pSNH2000	Ele	0.116	(-0.006, 0.234)	0.062

Figure S1. Rarefaction and extrapolation curves of different habitats based on true bug individuals. The solid portions of the four curves represent the actual total number of individuals and species sampled, while the dashed portions represent the predicted values for the number of individuals and species. When the dashed portions approach a horizontal trend, it indicates that sampling is relatively adequate. The shaded areas represent the confidence intervals. As evident from the figure, the sampling curves for the four habitat types are relatively flat, with the dashed lines at the tails approaching a horizontal trend, indicating that sampling of the bug communities in the four habitat types is relatively adequate. C: cultivated land; F: natural forest; P: planted forest; S: complex habitat.

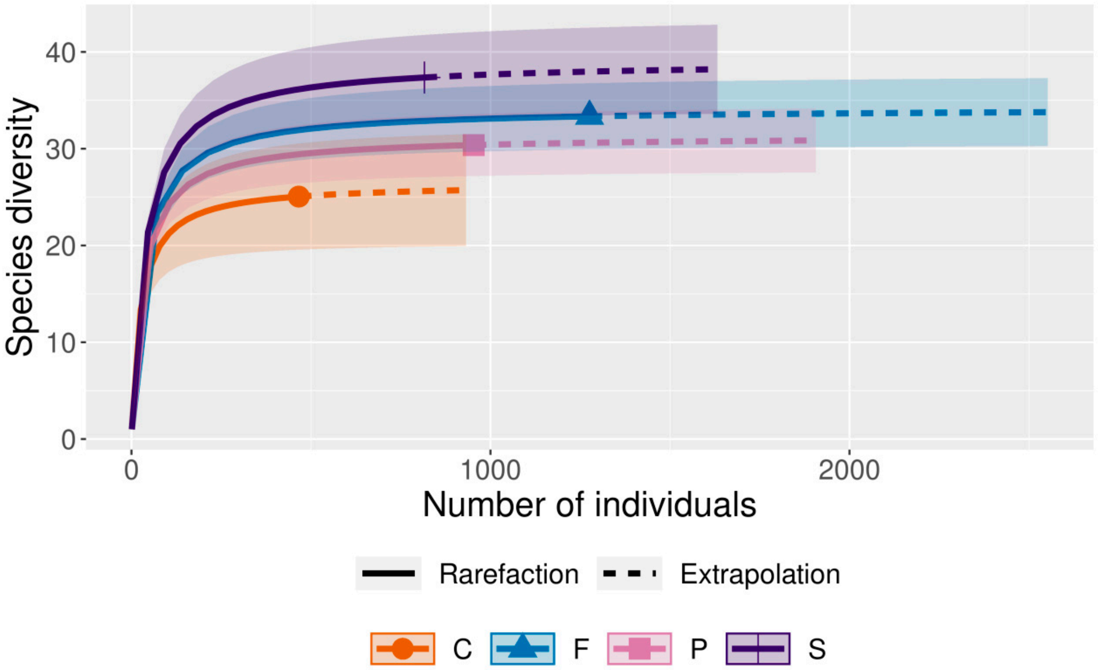

Figure S2. Comparative analysis of true bug functional diversity across different habitat types. C: cultivated land; F: natural forest; P: planted forest; S: complex habitat.

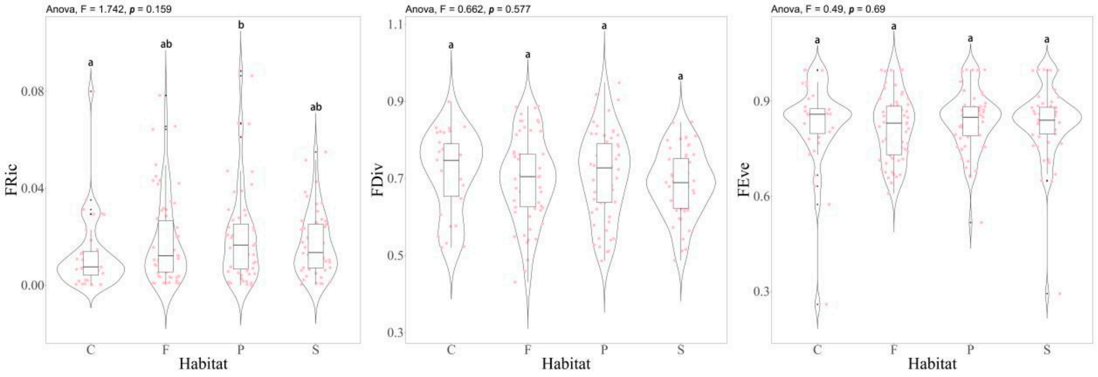

Figure S3. Influence of landscape variables on the richness of true bugs across 2000m, 3000m and 5000m spatial scales. Only significant effects are plotted (refer to Supplementary Table S6). C: cultivated lands, F: natural forest, P: planted forest, S: complex habitats. The numbers 2000, 3000

and 5000 represent the spatial scales of the landscape variables, measured in meters.

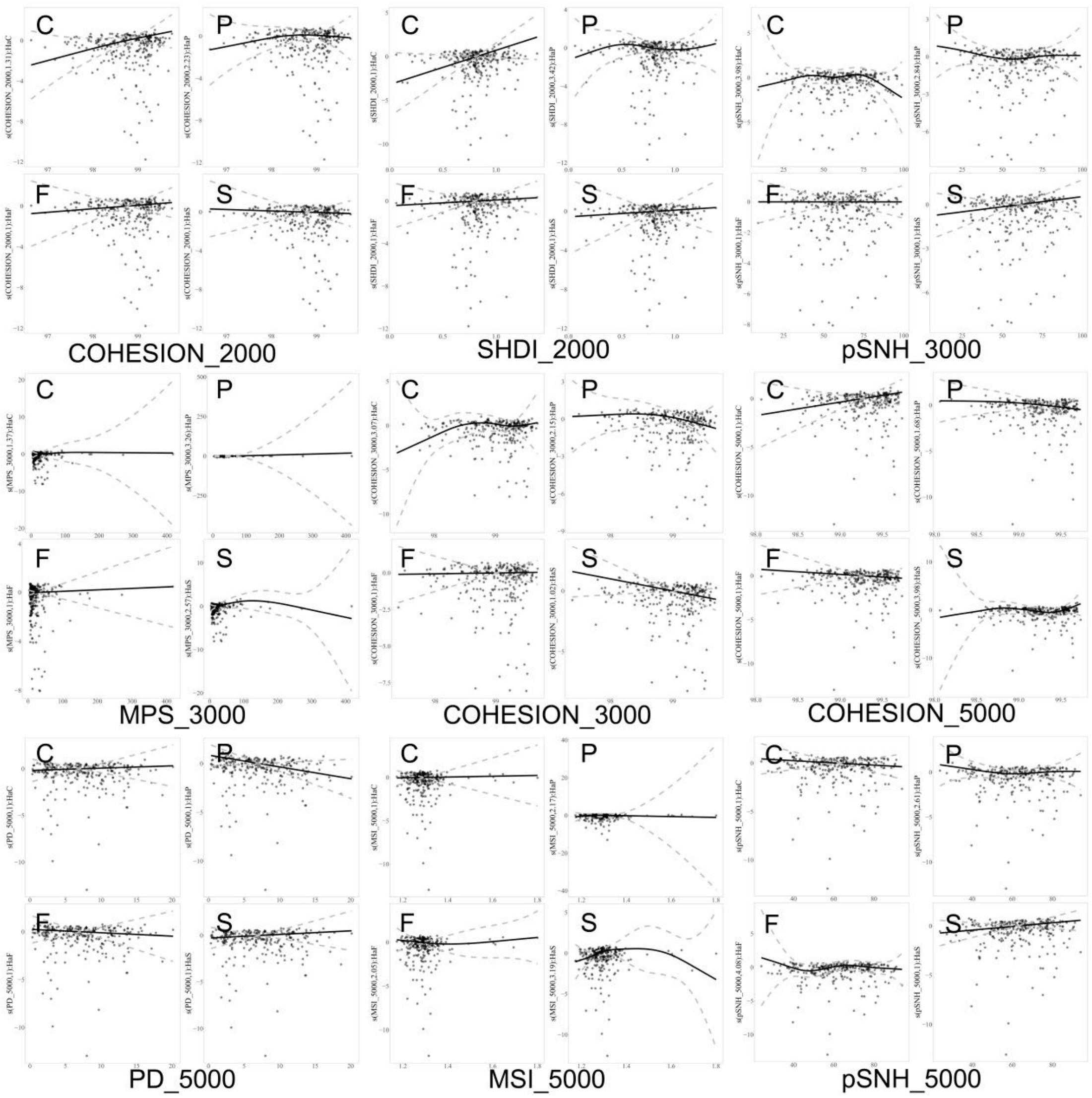

Figure S4. Influence of landscape variables on the abundance of true bugs across 2000m, 3000m and 5000m spatial scales. Only significant effects are plotted (refer to Supplementary Table S6). C: cultivated lands, F: natural forest, P: planted forest, S: complex habitats. The numbers 2000, 3000 and 5000 represent the spatial scales of the landscape variables, measured in meters.

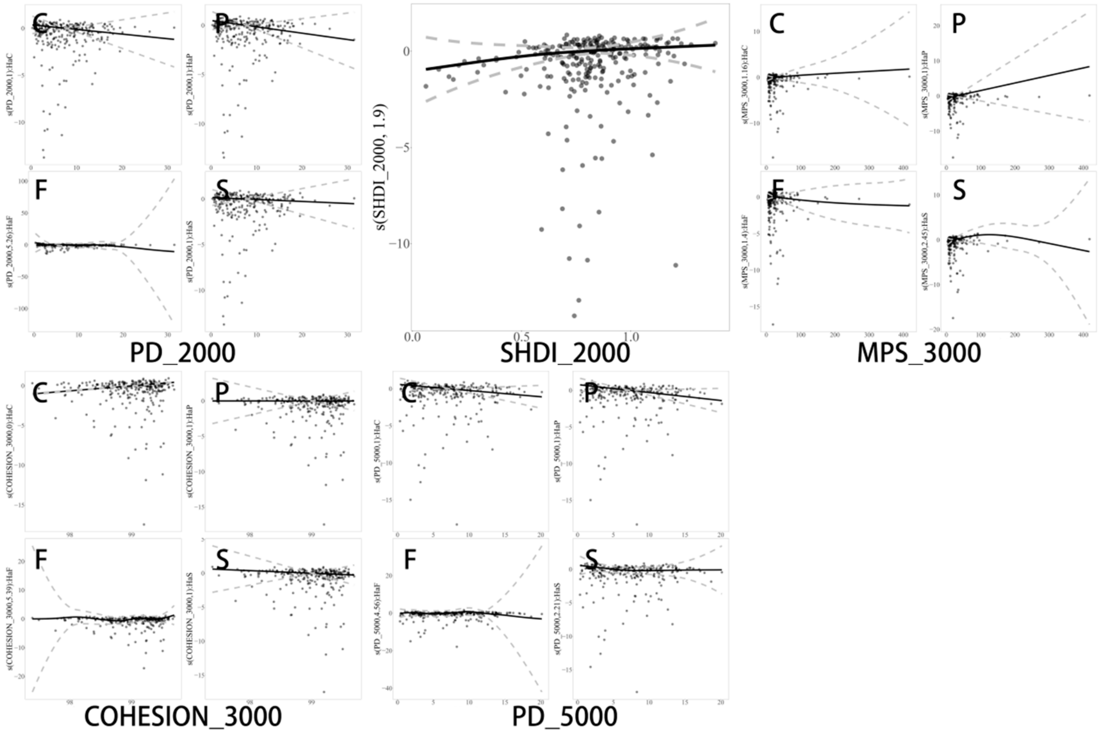

Figure S5. Influence of landscape variables on the functional richness of true bugs across 3000m and 5000m spatial scales. Only significant effects are plotted (refer to Supplementary Table S6). C: cultivated lands, F: natural forest, P: planted forest, S: complex habitats. The numbers 3000 and 5000 represent the spatial scales of the landscape variables, measured in meters.

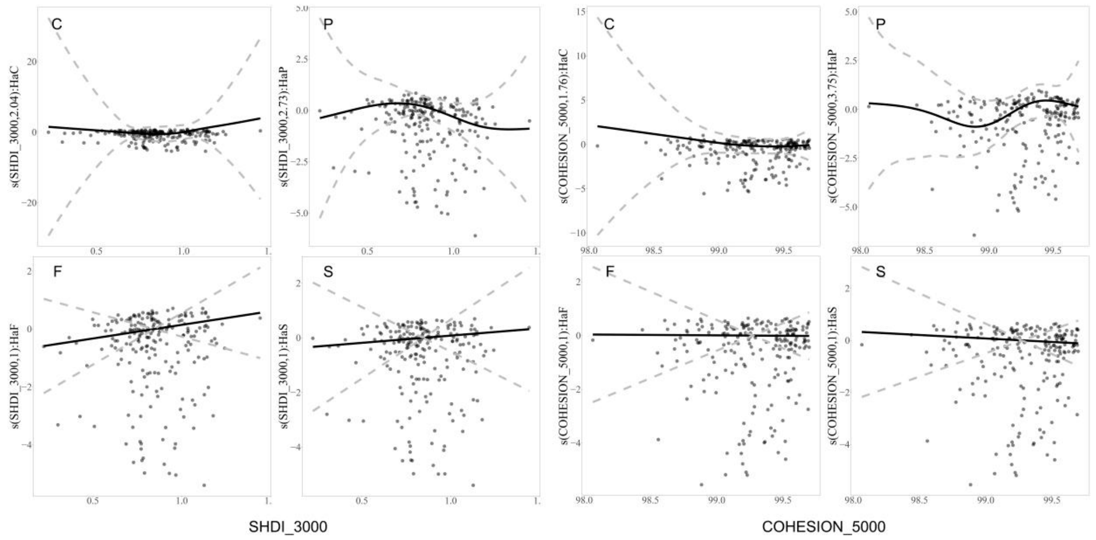

Figure S6. Influence of landscape variables on the functional evenness of true bugs across 3000m and 5000m spatial scales. Only significant effects are plotted (refer to Supplementary Table S6). C: cultivated lands, F: natural forest, P: planted forest, S: complex habitats. The numbers 3000 and 5000 represent the spatial scales of the landscape variables, measured in meters.

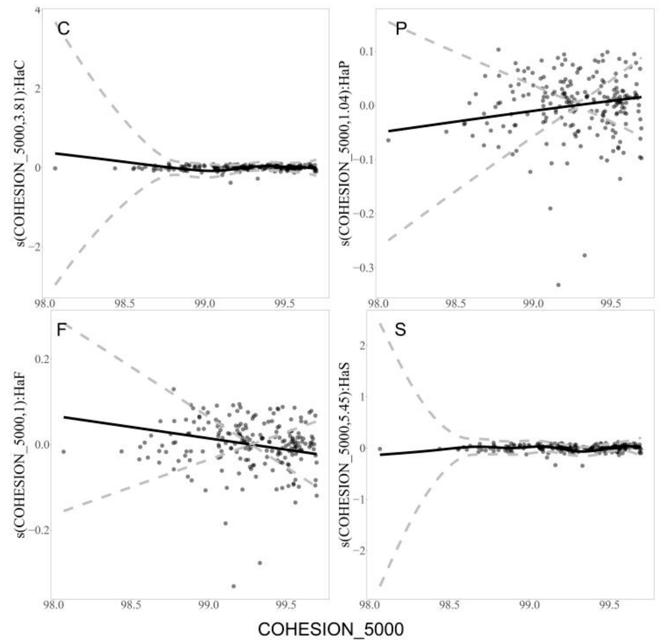

Figure S7. Influence of landscape variables on the functional divergence of true bugs across 3000m and 5000m spatial scales. Only significant effects are plotted (refer to Supplementary Table S6). C: cultivated lands, F: natural forest, P: planted forest, S: complex habitats. The numbers 3000 and 5000 represent the spatial scales of the landscape variables, measured in meters.

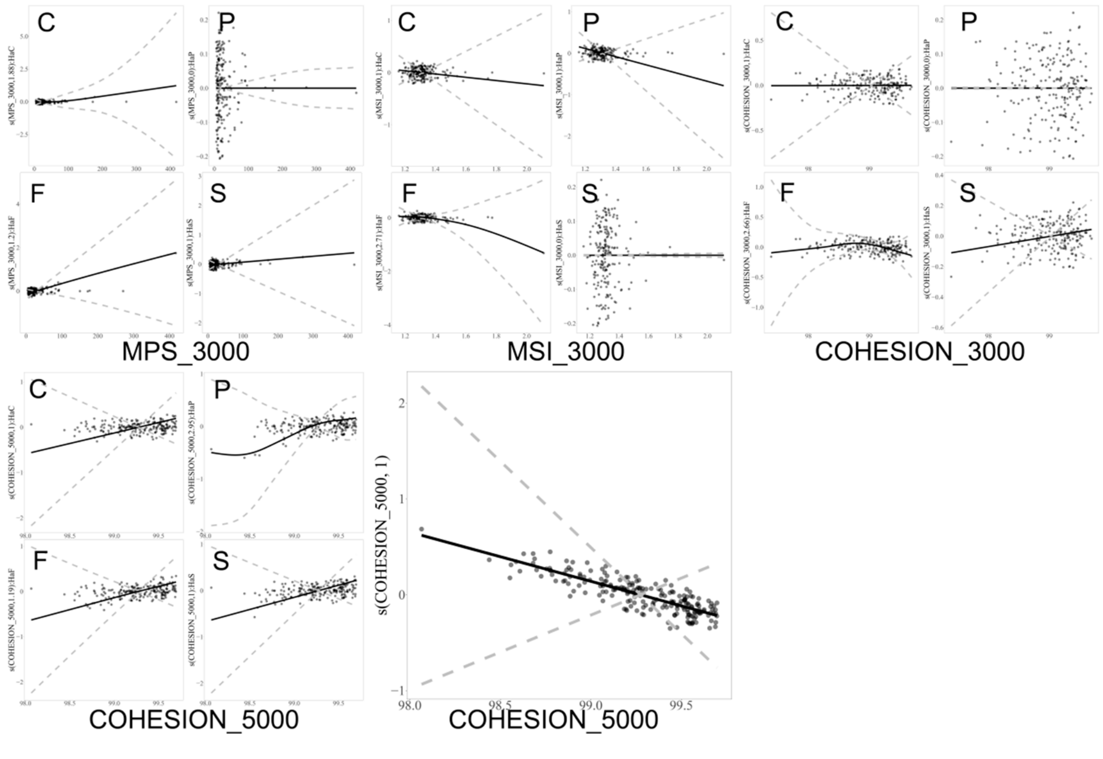

Figure S8. Pearson correlation analysis between species richness and functional richness, functional evenness, functional divergence. The lower left panel displays pairwise variable comparison plots, the diagonal features density curve plots, and the upper right panel shows

Pearson correlation coefficients with significance annotations. *: P < 0.05; **: P < 0.01; ***: P < 0.001. Corr represents the overall correlation. C: cultivated land; F: natural forest; P: planted forest; S: complex habitat. Different colors represent different habitats. Red: cultivated land; green: natural forest; blue: planted forest; purple: complex habitat. Across both overall and varied habitats, a significant positive correlation was observed between functional richness and species richness. However, no significant correlation was found between functional evenness and species richness. Notably, within natural forests alone, the functional divergence of true bugs exhibited a significant negative correlation with species richness.

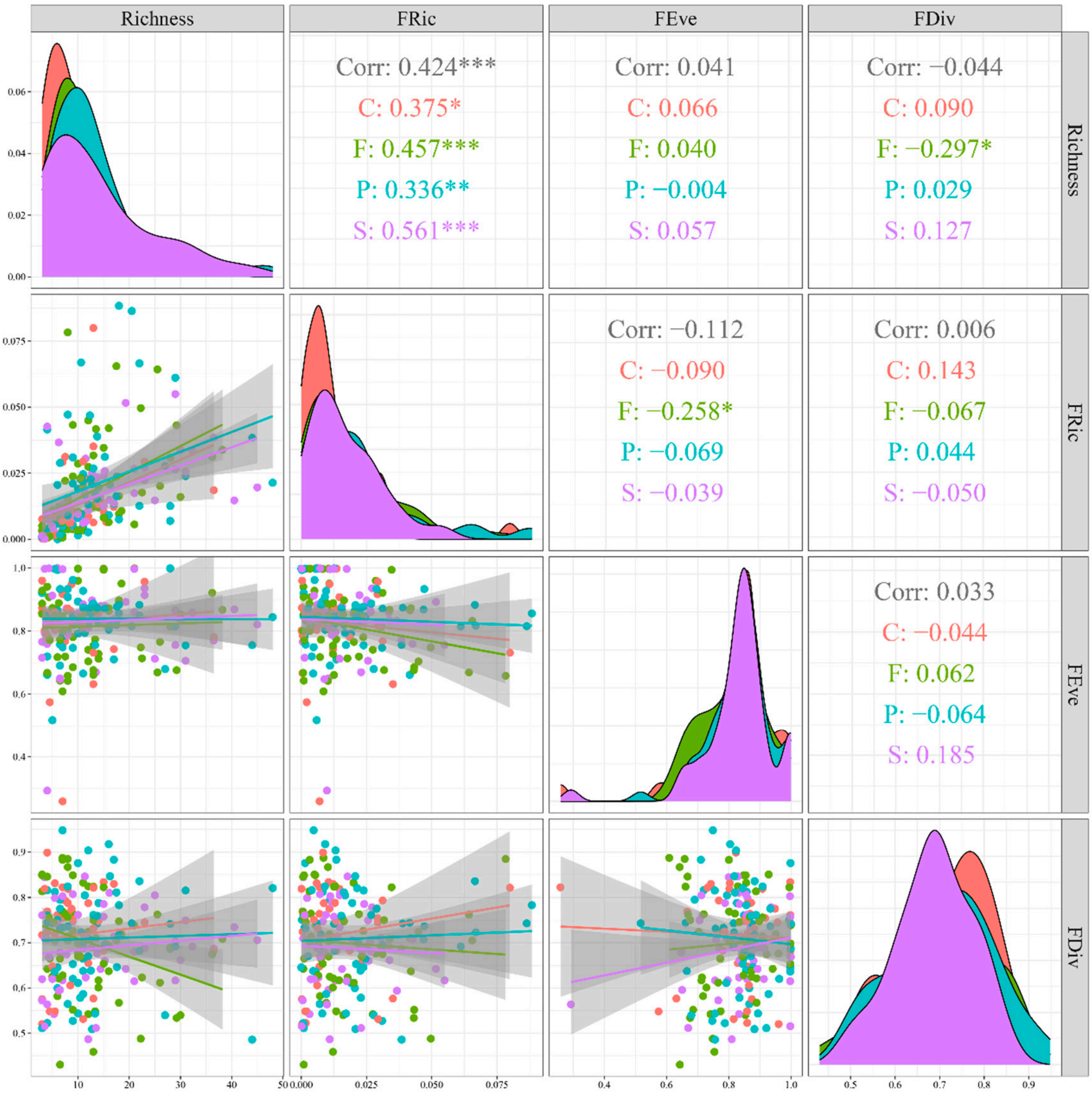

References

Gossner, M.M., Simons, N.K., Höck, L., Weisser, W.W., 2015. Morphometric measures of Heteroptera sampled in grasslands across three regions of Germany: Ecological Archives E096-102. Ecology 96, 1154–1154. https://doi.org/10.1890/14-2159.1

Figure S9. Diagnostic plots indicated generally satisfactory model performance under the Tweedie distribution. Residuals showed no strong systematic patterns against fitted values or linear predictors, and QQ-plots suggested an acceptable residual distribution.

(a) Species richness

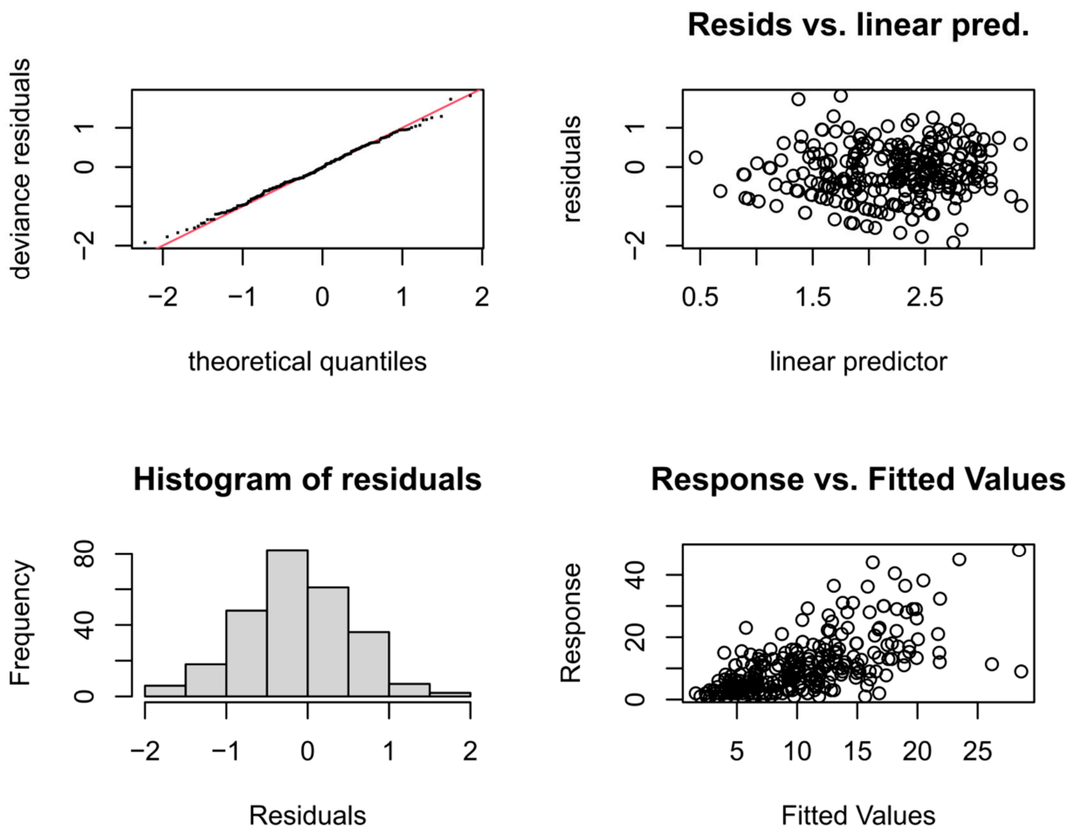

(b) abundance

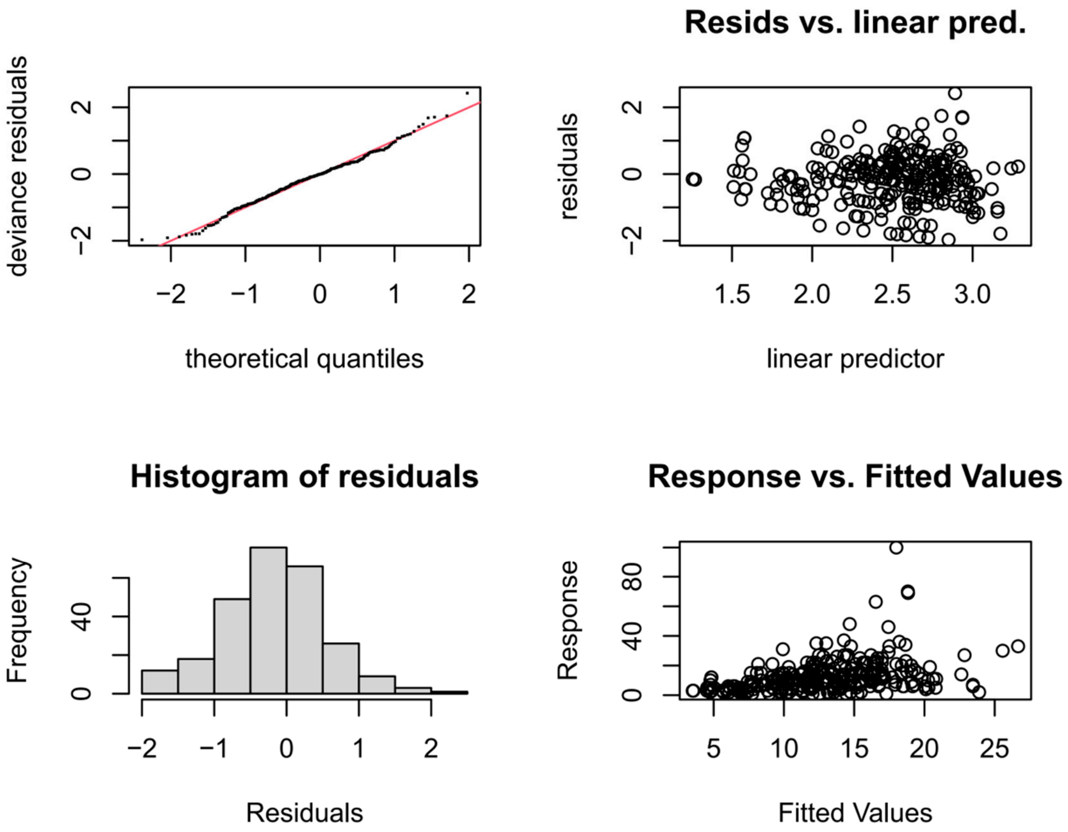

(c) Functional richness

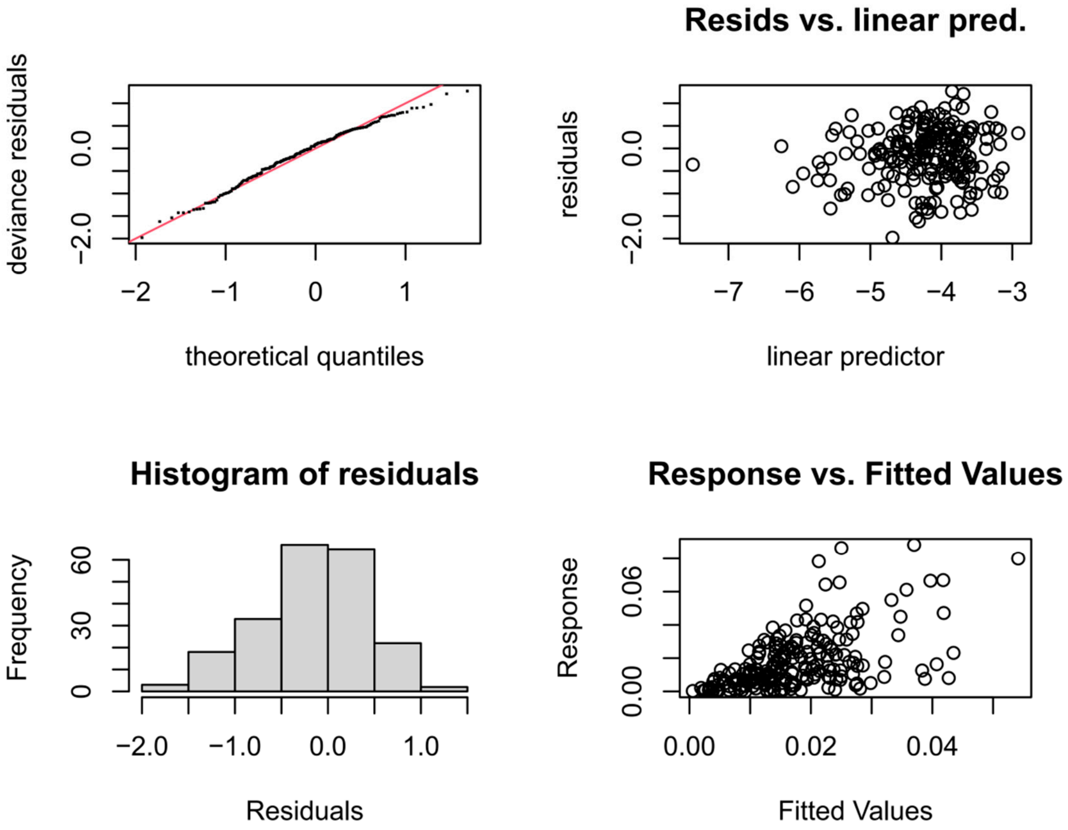

(d) Functional evenness

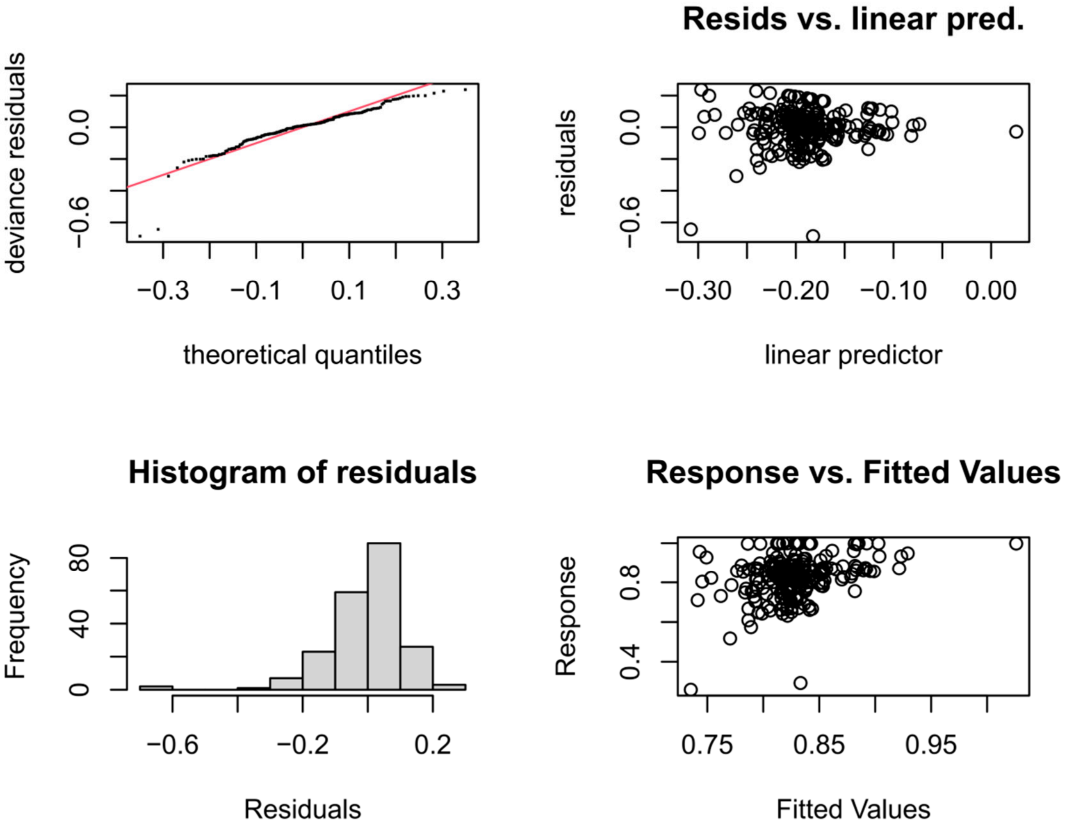

(e) Functional divergence

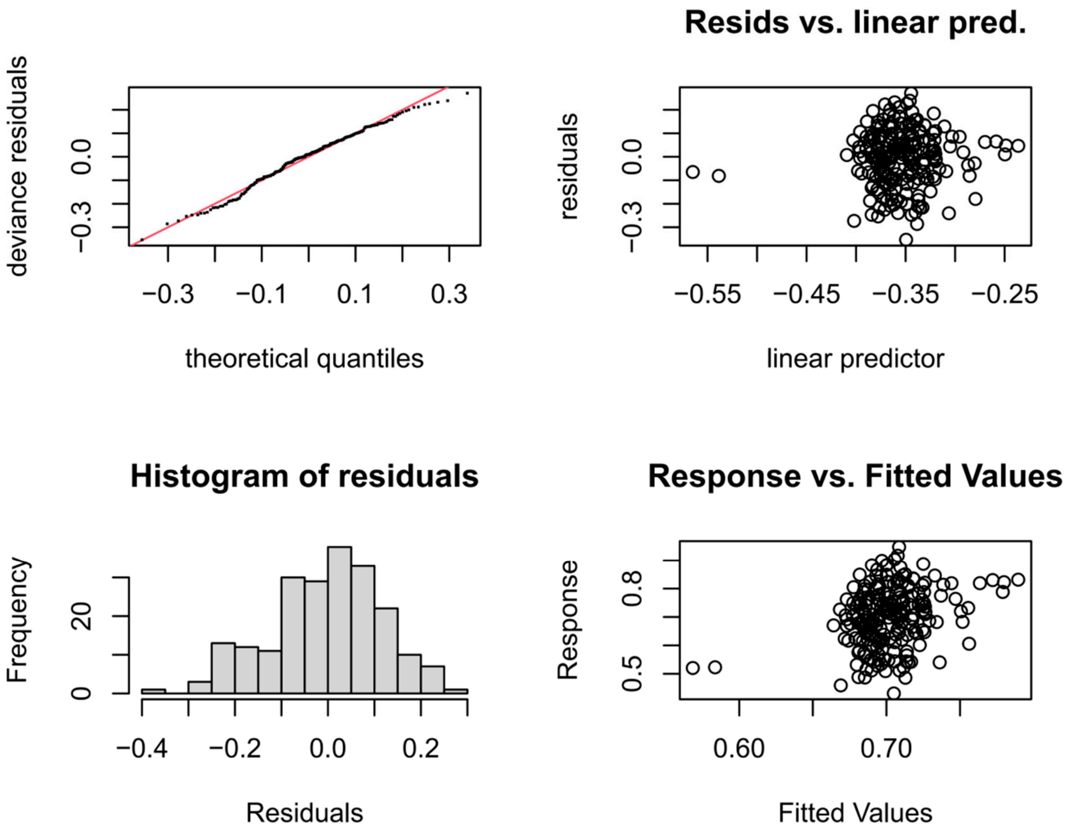
Resids vs. linear pred.
deviance residuals
theoretical quantiles
residuals
linear predictor
Histogram of residuals
Response vs. Fitted Values
Frequency
Residuals
Response
Fitted Values
